# Current Challenges and Future Directions in the Assessment of Glucocorticoid Status

**DOI:** 10.1210/endrev/bnae016

**Published:** 2024-05-25

**Authors:** Sophie A Clarke, Pei Chia Eng, Alexander N Comninos, Katharine Lazarus, Sirazum Choudhury, Christie Tsang, Karim Meeran, Tricia M Tan, Waljit S Dhillo, Ali Abbara

**Affiliations:** Section of Investigative Medicine, Imperial College London, London W12 ONN, UK; Department of Endocrinology, Imperial College Healthcare NHS Trust, London W6 8RF, UK; Section of Investigative Medicine, Imperial College London, London W12 ONN, UK; Department of Endocrinology, Imperial College Healthcare NHS Trust, London W6 8RF, UK; Department of Endocrinology, National University of Singapore, Singapore; Section of Investigative Medicine, Imperial College London, London W12 ONN, UK; Department of Endocrinology, Imperial College Healthcare NHS Trust, London W6 8RF, UK; Section of Investigative Medicine, Imperial College London, London W12 ONN, UK; Department of Endocrinology, Imperial College Healthcare NHS Trust, London W6 8RF, UK; Section of Investigative Medicine, Imperial College London, London W12 ONN, UK; Department of Endocrinology, Imperial College Healthcare NHS Trust, London W6 8RF, UK; Section of Investigative Medicine, Imperial College London, London W12 ONN, UK; Section of Investigative Medicine, Imperial College London, London W12 ONN, UK; Department of Endocrinology, Imperial College Healthcare NHS Trust, London W6 8RF, UK; Section of Investigative Medicine, Imperial College London, London W12 ONN, UK; Department of Endocrinology, Imperial College Healthcare NHS Trust, London W6 8RF, UK; Section of Investigative Medicine, Imperial College London, London W12 ONN, UK; Department of Endocrinology, Imperial College Healthcare NHS Trust, London W6 8RF, UK; Section of Investigative Medicine, Imperial College London, London W12 ONN, UK; Department of Endocrinology, Imperial College Healthcare NHS Trust, London W6 8RF, UK

**Keywords:** adrenal, pituitary gland, Addison's disease, steroids, glucocorticoid

## Abstract

Glucocorticoid (GC) hormones are secreted in a circadian and ultradian rhythm and play a critical role in maintaining physiological homeostasis, with both excess and insufficient GC associated with adverse effects on health. Current assessment of GC status is primarily clinical, often in conjunction with serum cortisol values, which may be stimulated or suppressed depending on the GC disturbance being assessed. In the setting of extreme perturbations in cortisol levels ie, markedly low or high levels, symptoms and signs of GC dysfunction may be overt. However, when disturbances in cortisol GC status values are less extreme, such as when assessing optimization of a GC replacement regimen, signs and symptoms can be more subtle or nonspecific. Current tools for assessing GC status are best suited to identifying profound disturbances but may lack sensitivity for confirming optimal GC status. Moreover, single cortisol values do not necessarily reflect an individual's GC status, as they are subject to inter- and intraindividual variation and do not take into account the pulsatile nature of cortisol secretion, variation in binding proteins, or local tissue concentrations as dictated by 11beta-hydroxysteroid dehydrogenase activity, as well as GC receptor sensitivity. In the present review, we evaluate possible alternative methods for the assessment of GC status that do not solely rely on the measurement of circulating cortisol levels. We discuss the potential of changes in metabolomic profiles, micro RNA, gene expression, and epigenetic and other novel biomarkers such as growth differentiating factor 15 and osteocalcin, which could in the future aid in the objective classification of GC status.

## Graphical Abstract

**Graphical Abstract:**
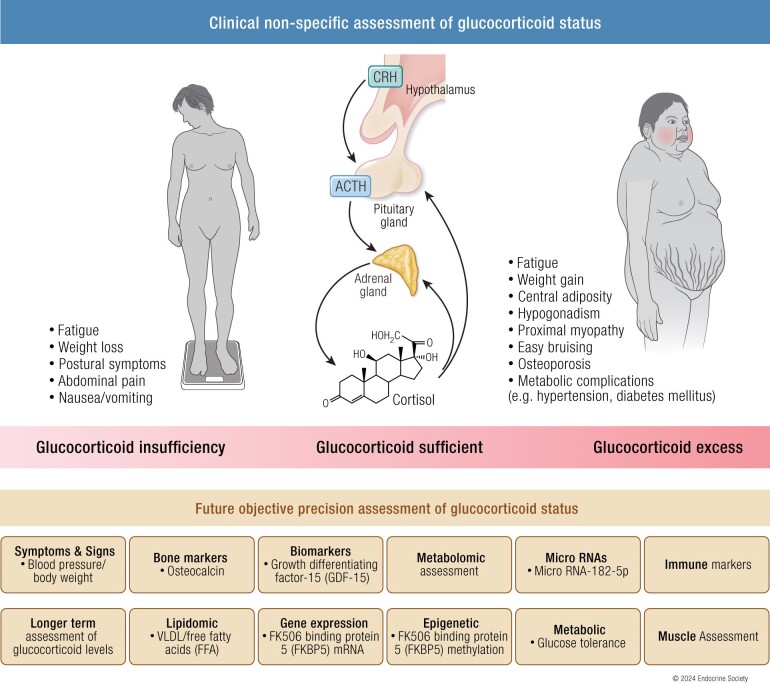

**Essential Points**

- Glucocorticoid (GC) hormones play a critical role in maintaining physiological homeostasis, with both excess and insufficient GC status being associated with adverse health outcomes.
- Current assessment of GC status employs relatively blunt tools aimed at identifying profound disturbances in adrenal function rather than confirming optimal GC status.
- Biomarkers that reflect cortisol action would improve the objective assessment of GC status.
- Putative biomarkers that could be altered by GC status include growth differentiating factor 15, metabolomic assessment, alteration in lipid/adipokines, micro RNA (eg, miR-122-5p), bone turnover markers (eg, osteocalcin), and epigenetic assessment (eg, FKBP5 methylation).

Glucocorticoid (GC) hormones are secreted in a circadian and ultradian pattern (1, 2) and are critical for maintaining physiological homeostasis (2). Both excess and insufficient GC levels are associated with adverse health outcomes (3, 4). However, symptoms and signs of GC dysfunction may only match GC status when perturbations are chronic and extreme, ie, markedly low or high cortisol levels.

Current assessment of GC status relies on clinical features in combination with measurement of cortisol levels, either in the basal state or stimulated/suppressed according to the GC disturbance being assessed. In addition, salivary and urinary cortisol may also be used. However, the accuracy of a single cortisol measure to reflect GC status is challenged by intra- and interindividual variation in cortisol concentrations, the pulsatile nature of cortisol secretion (5), variations in corticosteroid-binding globulin (CBG), and local tissue concentrations dependent on 11beta-hydroxysteroid dehydrogenase (11β-HSD) activity, as well as GC receptor sensitivity. Thus, several factors can influence individual sensitivity to GC (6).

Furthermore, GC status is not necessarily dichotomous (ie, either being unequivocally low or high), but rather there can exist a gradation in the degree of abnormality between insufficient and excess GC status, challenging confirmation of GC status. Moreover, GC status could be incongruent from measured cortisol levels or challenging in the setting of (1) concomitant use of exogenous steroid treatments (eg, inhalers or creams) that could suppress endogenous cortisol levels but still contribute to GC status; (2) assessment for Cushing's syndrome (CS) with incongruent results of investigations (eg, due to factors such as altered renal function affecting reliability of 24-hour urine collections); (3) early presentation of adrenal insufficiency (AI) with residual adrenal function whereby cortisol levels are not absent and cortisol response to stimulation tests may still be normal; (4) suboptimal GC replacement regimens whereby signs and symptoms of GC deficiency/excess can be nuanced; (5) “non-neoplastic hypercortisolism” whereby abnormal investigations may be equivocal/misleading; (6) acute illness whereby cortisol metabolism/clearance and levels are altered; (7) concomitant use of medications that could alter liver metabolism of GC (eg, affecting the response to a dexamethasone suppression test); (8) serum cortisol levels that are falsely elevated (eg, due to increased CBG as a result of oral estrogen treatment).

Herein, we discuss the challenges in the diagnosis/management of GC deficiency/excess and aim to evaluate novel markers that could be used in the objective assessment of GC status in the future that do not rely solely on the measurement of circulating cortisol levels (Fig. 1).

**Figure 1.**
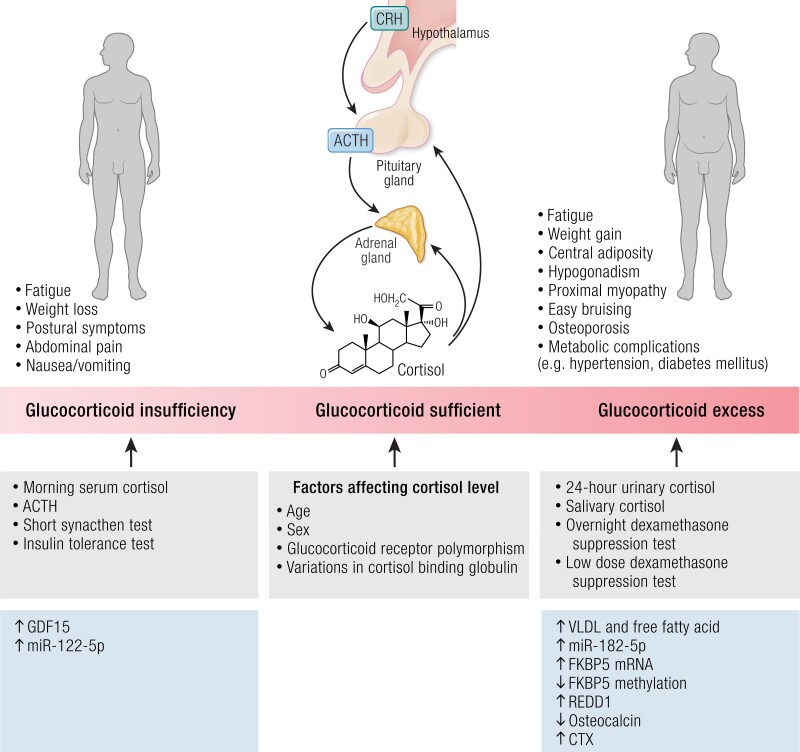
Factors affecting GC secretion, clinical factors affecting GC secretion, the clinical characteristics of an individual presenting with GC excess and GC depletion, and the investigations that a clinician can carry out to determine GC adequacy.

Abbreviations: CTX, C terminal telopeptide of type 1 collagen; FKBP5, FK506 binding protein 5; GC, glucocorticoid; miR-122-5, microRNA-122-5; miR182-5p, microRNA182-5p; REDD1, regulated in development and DNA damage response 1.

## The Hypothalamic-pituitary-adrenal Axis in Health

In healthy people who sleep at night, cortisol is released in both an ultradian (1 to 2 hourly cortisol pulses) (1) and circadian rhythm rising from ∼04:00 Am (7), peaking within 3 hours after waking (cortisol awakening response), before subsequently declining and reaching a nadir at around midnight (7). The frequency and amplitude of cortisol secretion are crucial to ensure synchronization of endogenous cellular rhythms to external cues (8). Clock genes in peripheral cells are responsible for circadian timekeeping with the activity of the suprachiasmatic nucleus and are modulated in the presence of external cues (eg, light, feeding, temperature) (8). Notably, acetylation of GC receptor by CLOCK/BMAL1 transcription factors is increased in the morning, leading to decreased cortisol tissue sensitivity (9).

The diurnal rhythm of circulating cortisol concentrations plays an important role in sleep timing and distribution of sleep stages throughout the night (10). However, the relationship is bidirectional with sleep onset and sleep disruption also influencing the release of cortisol (10). Sleep onset inhibits the hypothalamic-pituitary-adrenal (HPA) axis, typically within the first 20 minutes after slow-wave sleep with cortisol reaching a low level in the first half of the night (10). In the second half of the night, rapid eye movement sleep predominates, and inhibitory mechanisms are attenuated, which is associated by an increase in HPA activity (10). Nocturnal awakenings at the end of sleep are associated with transient release of cortisol, followed by temporary inhibition of cortisol secretion (10). Acute changes in the sleep-wake cycle (such as shiftwork or jetlag) lead to HPA axis activation and alter the normal circadian pattern of cortisol secretion (10). Some (11), but not all (12), chronic shift workers are reported to have a delay in the early morning cortisol rise. Prolonged exposure to high cortisol levels is associated with reduced glucose tolerance and insulin sensitivity (13).

Currently, disturbance of GC status is assessed using basal or stimulated/suppressed cortisol levels depending on the GC disturbance being assessed (Fig. 1). Moreover, ACTH is pulsatile and variable such that it may not be reliable as a marker of GC status in isolation especially if the disturbance in GC status is not at the extreme end of being very high or very low (2).

## Factors Affecting Circulating Cortisol Concentration

Measurement of cortisol concentrations can be affected by several factors including differences in age, sex, time of collection, assay factors, and variation in binding protein levels.

### Variation in Corticosteroid Binding Globulin

CBG is predominantly produced by the liver and has a high affinity for cortisol (14). Approximately 90% of serum cortisol is bound to CBG, 4% to 6% is bound to other binding proteins (eg, albumin or alpha-1-glycoprotein), and 4% to 6% circulates freely (14, 15). Cortisol binding to CBG is a major determinant of bioavailability; thus, interindividual variability of binding to CBG may affect the measurement of total cortisol levels and dynamic tests (14, 15), although salivary cortisol and urinary free cortisol are not affected by alterations in CBG (16). Synthetic GCs, including prednisolone and dexamethasone, bind CBG with less affinity than cortisol (15, 17). Other factors affecting CBG include sex, temperature, glycosylation states, critical illness, genetic variants, medications, and insulin sensitivity (14, 18).

Of clinical relevance, low albumin states (albumin < 25 g/L), as encountered in critical illness, are associated with lower total cortisol levels (435 vs 633 nmol/L) but similar active free cortisol levels (140 vs 143 nmol/L) (19). Likewise, nephrotic syndrome results in renal CBG loss and thus lower total cortisol levels and thus could lead to a false diagnosis of AI after a tetracosactide test (tetracosactide is amino acid 1-24 of the 36 amino acid structure of ACTH, commonly marketed as Synacthen®) (20). Conversely, exogenous oral estrogens (as well as high circulating levels of estrogens in pregnancy) increase CBG, resulting in a falsely elevated total cortisol (21).

### Age and Cortisol Secretion

Age influences endogenous cortisol secretion and diurnal rhythmicity; increasing age leads to a reduction in the diurnal amplitude of cortisol secretion (22, 23), an advancement of the early morning cortisol rise (24), a reduction in the amplitude of cortisol pulses, and an increase in serum cortisol nadir. However, age-specific cut-offs are typically not used in clinical practice.

### Effect of Sex and the Menstrual Cycle

Cortisol levels vary between men and women, with additional differences due to the menstrual cycle and menopause (25). Men have higher cortisol output with higher mean peak, nadir, and cortisol awakening response than premenopausal women (26, 27). The peak cortisol rise (acrophase) increases gradually with age in women but not in men, such that there is no longer a difference after menopause (25).

Studies evaluating cortisol responses at different stages of the menstrual cycle have shown discordant results. Some studies have not shown any difference in mean salivary cortisol levels across the menstrual cycle (28, 29), while other studies have shown higher circulating cortisol levels in the follicular phase (30). A higher stimulated cortisol response following tetracosactide has been reported in the luteal phase (29, 31). Such factors could cause a discrepancy between cortisol levels and GC action, and thus markers of GC action could more accurately reflect GC status in these situations.

## Single Cortisol Value in Reflection of GC Status

The variation in plasma cortisol levels with time-of-day challenges interpretation of a single value taken at a random time. Early morning cortisol levels are generally high, and levels in patients who are asleep at midnight are usually very low. A major challenge in the interpretation of serum and stimulated cortisol levels is the lack of assay-specific normative data in specific population groups (32, 33). Historically, serum cortisol was measured using a nonspecific fluorometric assay that measures both cortisol and corticosterone (34). Due to the poor specificity and low sample throughput of this method (35), cortisol measurement commonly is undertaken using automated cortisol immunoassays (36) with each clinical laboratory establishing its own performance characteristics and specificity for cortisol (33). Agreement between different immunoassays vary (37); according to some studies, levels can be comparable to those of mass spectrometry (38), while others have reported higher cortisol levels using immunoassay than mass spectrometry (39–41). A study comparing automated immunoassays [Advia Centaur (Siemens), Architect (Abbott), Modular Analytics E170 (Roche), Immulite 2000 (Siemens), and Access (Beckman)] with gas chromatography-mass spectrometry showed a bias of 1.08 to 1.36 in immunoassay-measured cortisol levels (32). In addition to the analytical bias among immunoassays, sex-specific normative cortisol responses in healthy individuals remain undefined (33). Direct cortisol immunoassays may be susceptible to cross-reactivity with other structurally similar compounds, in particular prednisolone and 11-deoxycortisol (42).

This difference among cortisol immunoassays ultimately affects the interpretation of GC status (42). The discrepancy between serum cortisol levels measurement between mass spectrometry and automated immunoassays (12.6% to 90.8%) can be greater in patients receiving treatment for CS e.g. metyrapone owing to the cross-reactivity of structural cortisol analogs in the steroidogenesis pathway (43). Mass spectrometry quantifies exogenous GC (44) and endogenous GC metabolites (45) simultaneously (43), and recent developments have improved sensitivity and reliability (46), allowing novel insights into GC processing to be investigated, although mass spectrometers are not ubiquitously available (47).

In view of the various factors that interfere with cortisol levels, measurement of serum-free cortisol (5% of total) was proposed to have better clinical utility in patients with total cortisol concentrations near the diagnostic threshold or in states of altered binding globulin (16). Free cortisol can be calculated after removing bound fractions via a suitable technique either by ultrafiltration, equilibrium dialysis, or gel-filtration chromatography (39, 48). Although these techniques produce comparable interassay coefficients of variation < 10% (49), they are labor-intensive and require long incubation times. Surrogate methods (eg, Coolen's equation) or the free cortisol index have been used to estimate free cortisol levels (50), but these equations are limited in their assumption of direct proportionality and can be inaccurate in patients with hypoalbuminemia or altered CBG capacity (51, 52).

Thus, the validity of using cortisol response to tetracosactide test to diagnose AI can be influenced by assay performance and alterations in CBG (32, 33). The widely used cortisol cut-offs of 500 (53) or 550 nmol/L (54) in response to a tetracosactide test could result in misclassification of AI (12%,19%, 4%, and 9%) for the Centaur, Architect, Immulite (2000), and Access assays, respectively, at 500 nM; 27%, 42%, 16%, and 21% for the respective assays at 550 nM (32), whereas patients with a morning (8:00 Am to 12:00 Pm) serum cortisol (using an ultrasensitive immunoassay on an Abbott Architect platform) of >275 nmol/L (96% sensitivity) are unlikely to have an insufficient response to tetracosactide (55).

Moreover, GC insufficiency can exist over a continuum between complete lack of cortisol and partial deficiency/inability to mount an adequate stress response, and conversely cortisol values marginally below the lower reference limit may not necessarily reflect GC inadequacy. Therefore, other parameters reflecting GC action could provide useful information in addition to clinical judgment in the assessment of GC status.

## Current Assessment of Altered GC States

### GC Insufficiency

AI is characterized by a failure to secrete sufficient GC to meet physiological demands. Primary adrenal insufficiency (PAI) accounts for 40% of AI (56), resulting from destruction of adrenal cortical cells (typically due to an autoimmune process or infection, eg, tuberculosis), or adrenal enzymatic failure of GC synthesis (eg, congenital adrenal hyperplasia) (4, 57). Secondary adrenal insufficiency (SAI) occurs due to impaired ACTH production, eg, due to a pituitary adenoma or traumatic brain injury (56). Tertiary AI occurs due to impaired hypothalamic corticotrophin releasing hormone secretion, usually due to chronic exogenous GC use (4). In patients with AI, GC replacement is used to avoid the life-threatening complication of adrenal crisis and to optimize well-being (58). Patients with PAI also usually require additional mineralocorticoid therapy (59).

#### Clinical and biochemical assessment of GC insufficiency

Symptoms of AI depend on the degree of cortisol, mineralocorticoid, and adrenal androgen deficiency at the time of presentation and can be nonspecific, which can lead to delayed diagnosis (60). Clinical presentation and biochemical changes seen in patients with AI are presented in Table 1 and of GC excess in Table 2. Dynamic endocrine tests used in the diagnosis of both GC deficiency and GC excess and their associated challenges are summarized in Table 3.

**Table 1.** Clinical features in AI

Clinical features	Prevalence in AI (%)	References
Fatigue	95	61
Weight loss	70-100	62
Decreased appetite	62	62
Nausea	57	63
Vomiting	52	63
Postural symptoms	56	64
Salt-craving	38-64	65
Abdominal pain	22	63
Skin darkening (including buccal pigmentation)	74	65
Biochemical changes		
Hyperkalemia	40	66
Hypercalcemia	10-20 of acute crises	61, 67
Hyponatremia	70-80	62, 63

Abbreviations: AI, adrenal insufficiency.

**Table 2.** Clinical features of Cushing's syndrome

Clinical features	Prevalence in Cushing's syndrome (%)	References
Nonspecific symptoms and signs		
Muscle weakness	40-70	68
Cognitive and psychiatric changes	50-81	69
Reduced libido	47	70
Sleep disturbance and fatigue	60	71
Menstrual disturbances (due to inhibition of GnRH)	56	70
Hair loss	31	70
Obesity and recent weight gain	70-95	72
More objective clinical features		
Changes in fat deposition (increased fat around face, supraclavicular, and dorsocervical regions)	50-90	73, 74
Violaceous striae	44-50	72
Proximal myopathy	67	70
Easy bruising	35-65	69, 72
Hirsutism	56-75	70
Complications of Cushing's syndrome		
Diabetes mellitus	20-47	75
Impaired glucose tolerance	45-70	72
Osteopenia	60-80	69, 70
Bone fractures at any sites	42	
Osteoporosis	31-50	69, 75
Bone fractures at any sites	62	
Atherosclerotic changes	27-31	68
Hypertension	58-85	75
Nephrolithiasis	50	75
Biochemical changes		
Hypokalemia	42-70 in ectopic ACTH, 10 in Cushing's disease	76
Dyslipidemia	38-71	75

**Table 3.** Challenges in interpretation of the results of tests used in the assessment of GC status

Biochemical test	Current challenges in the assessment of GC status
GC deficiency (adrenal insufficiency)	
Morning serum cortisol	Cortisol cut-off of <140 nmol/L suggestive of AI (77) Challenge in establishing normal ranges—a cortisol value of 140 nmol/L is close to the lower limit of the range of healthy individuals (78) Assay specific cut-off values will vary between different methods to evaluate cortisol levels (32)
Short tetracosactide test (Synacthen®) (250 mcg)	Precise thresholds dependent on local assays (79), but using more specific cortisol assays utilizing LC-MS/MS, cortisol cut-off of 14-15 µg/dL (386-413 nmol/L) is recommended to avoid false-positive results (80) Clinical variability as to whether 30- or 60- minute cortisol values are used (81) Other factors, eg, changes in cortisol binding to CBG, and factors affecting CBG will all impact results (14)
ITT	Need for adequate hypoglycemia (2.2 mmol/L) to fully interpret the cortisol value and requires intensive medical and nursing supervision (82) Contraindicated in certain patient populations, including epilepsy and cardiac disease (82) Need to stop oral estrogens 6 weeks before the test due to effect on CBG (14)
Glucagon stress test	Assay-specific threshold value Sensitivity 71% and specificity 57% with a cortisol cut-off value >350 nmol/L (using the Abbott Architect assay), when compared with the metyrapone suppression test (83) To use with caution as a test to assess the HPA axis where an ITT is contraindicated
Metyrapone suppression test	Validated against the ITT for the diagnosis of secondary hypoadrenalism Sensitivity of 47% and specificity 82% with 11-deoxycortisol <200 nmol/L; this increases to a sensitivity of 71% and specificity of 69% when used with a cortisol value of 450 nmol/L (84)
GC excess (Cushing's syndrome)	
Low-dose dexamethasone suppression test	Sensitivity 80%-95% (85, 86) Specificity 80%-95% (dependent on cut-off value) (85, 86) Affected by dexamethasone clearance and metabolism—including medications affecting CYP3A4 complex, eg, rifampicin (87) Affected by changes in CBG, eg, use of oral estrogens (increasing CBG) and nephrotic syndrome/cirrhosis (reducing CBG) (14)
24-hour UFC measurement	Sensitivity 45%-71% (72) Specificity up to 100% (88) Need for >1 test to avoid false-negative results and in the detection of cyclical Cushing's (89) Variable UFC in individuals with cortisol excess—ranging from normal to severely elevated including raised levels in nonneoplastic hypercortisolism (90) Affected by assay type—risk of cross-reactivity with cortisol precursors and metabolites in immunoassays compared with HPLC or tandem mass spectrometry leading to discordant results between assay types (91) Risk of falsely raised results with >5L urine collection and falsely low with a reduced eGFR (92, 93)
Late-night salivary cortisol	Sensitivity 92% to 100% (94, 95) Specificity 85% to 100% (94, 95) Increases with age, hypertension, and diabetes and in shift workers Risk of false positives with immunoassays (94, 95)

In the diagnosis of Cushing's syndrome, a diagnosis was established on a composite of clinical findings and other concordant investigations.

Oral oestrogens increase CBG and therefore should be stopped 6 weeks prior to tests measuring cortisol as an endpoint.

Abbreviations: AI, adrenal insufficiency; CBG, corticosteroid-binding globulin; GC, glucocorticoid; eGFR, estimated glomerular filtration rate; HPLC, high-performance liquid chromatography; ITT, insulin tolerance test; LC-MS/MS, liquid chromatography-mass spectrometry; UFC, urinary free cortisol.

#### Assessment of GC status in AI with replacement therapy

Patients with AI, including both primary and secondary, have increased mortality rates (96), attributed to excess GC exposure at nonphysiological times and adrenal crises (97). GC excess is associated with metabolic dysfunction, including hypertension, dyslipidemia, immune suppression (98), hyperglycemia, and osteoporosis (3). Optimizing replacement therapy for AI, using multidose oral regimens, therefore involves minimizing excess GC exposure and replicating the circadian rhythm (99); however, to date, there is a paucity of universally accepted methods to ascertain optimal GC replacement. In patients receiving multiple daily-dose hydrocortisone regimens, hydrocortisone day curves can be used predominantly to assess interindividual pharmacokinetic alterations in hydrocortisone metabolism (58). For those taking low-dose prednisolone (eg, 2-4 mg once daily), mass spectrometry can quantify prednisolone concentrations as low as 10 mg/L (100), enabling higher resolution day curves with the potential to individualize dosing regimens (101).

A study evaluating a novel GC replacement regimen highlighted potential measurable pharmacodynamic differences that could be monitored to assess GC status (99, 102). Once-daily modified release hydrocortisone (dose 16 mg/m^2^) compared with standard hydrocortisone/cortisone acetate (dose 18 mg/m^2^) showed differences in body weight, immune profiles, and quality of life scores (101). Modified-release hydrocortisone was associated with more physiological immune profiles [reversal of the increased number of CD16+ natural killer (NK) cells at baseline] and reduced susceptibility to infections (101, 103). Similarly, studies evaluating cortisol replacement via pulsatile administration pumps have used functional imaging, quality of life scores (SF-36), and behavioral and cognitive tests to assess GC effects on emotional processing (104).

### GC Excess

#### Clinical and biochemical assessment of GC excess

Many symptoms of CS are common but nonspecific, while others are more specific but less common (68). This can be complicated by associated comorbidities such as obesity and hypertension that are commonly found in the general population, as well as sex, age (105), and the duration and extent of disease (106). Table 2 summarizes the clinical features and complications observed in CS. More discriminatory features relate to the catabolic state and increased protein breakdown observed secondary to GC excess (68). The use of facial recognition to diagnose CS is yet to be validated in a clinical setting, but face-classification software correctly classified 85% of patients and 95% of controls (68). Table 3 outlines 3 biochemical screening tests recommended by the Endocrine Society, where there is a strong pretest probability of a diagnosis of CS (89). However, the sensitivity and specificity of the tests will depend on the assay and threshold values used. There can also be intraindividual variability, and more than 1 measurement, eg, in the case of urinary free cortisol, is often required to avoid false-negative results (90). Moreover, in the setting of cyclical CS, results may be concordantly negative and require further evaluation and follow-up (89). Other methods of measuring cortisol levels include salivary cortisol, whereas hair and nail cortisol could be used to reflect longer term GC exposure.

### Salivary Cortisol and Cortisone

Unconjugated cortisol diffuses into the saliva and is converted to cortisone by the enzyme 11β-HSD type 2 (11β-HSD2) (107). Serum cortisone levels are 4-fold lower than serum cortisol levels, whereas salivary cortisone levels are 6-fold higher than salivary cortisol levels (ratio of salivary cortisone: salivary cortisol = 6:1) (108). Salivary cortisol and cortisone showed good correlation with serum free cortisol (108). However, in a study of women treated with estrogens and healthy controls (HCs), salivary cortisone correlated more strongly with serum cortisol levels (Spearman *r* = +0.95) than salivary cortisol (109). High levels of salivary cortisol were found for ∼45 minutes after oral dosing of hydrocortisone, likely due to detection of residual hydrocortisone in the oral cavity (109). However, as hydrocortisone pills do not contain cortisone, salivary cortisone measurement is not affected by oral hydrocortisone, and its level remains correlated with serum free cortisol (*ρ* = 0.91) in healthy male volunteers (n = 14) after both oral or intravenous hydrocortisone administration (110). Salivary cortisone is more readily measurable, remaining detectable across the whole 24-hour day, whereas salivary cortisol concentrations can fall below detection limits at low serum cortisol levels (110).

A recent prospective study demonstrated that waking salivary cortisone measured by liquid chromatography-tandem mass spectrometry could predict cortisol response >430 nmol/L at 30 minutes following tetracosactide (measured by immunoassay) with an area (AUC) of 0.95 [95% confidence interval (CI) 0.92-0.97] (111). This was greater than the performance of waking salivary cortisol (AUC 0.89, 95% CI 0.85-0.94), which is more susceptible to contamination from oral hydrocortisone from the previous day in a small number of patients (111). This approach could avoid the need for tetracosactide stimulation in 70% of patients (111).

Late-night salivary cortisol and cortisone measured by liquid chromatography-tandem mass spectrometry both have high specificity and sensitivity for identifying patients with endogenous CS (110). A threshold for late-night salivary cortisone of >14.5 nmol/L had a sensitivity of 95.2% and specificity of 100% for identification of CS (112). Although liquid chromatography-mass spectrometry offers better specificity and comparable sensitivity than antibody-based methods, it is not widely available (113).

Therefore, salivary cortisone can be used in the screening for AI to assess cortisol circadian rhythm in patients treated with oral hydrocortisone (110) or to evaluate hydrocortisone replacement in children where blood sampling is undesirable (114). Three 8-hourly salivary cortisone measurements provided a good estimate of AUC cortisol exposure in HCs (n = 28), patients with AI, or autonomous cortisol secretion (n = 8) and could be used to assess 24-hour cortisol secretion (115).

Saliva collection is easy for most adults but can be challenging for infants, elderly patients, patients with oral lesions, or patients with Sjogren's syndrome (113). Immunoassays used to analyze salivary cortisol/cortisone are inexpensive to run, but there is a potential of cross-reactivity with other steroids, and they are generally not validated for the salivary matrix at the concentrations required (91, 116, 117).

### Hair Cortisol

Hair cortisol analysis has been investigated in the research setting as a potential tool for the assessment of chronic GC excess (118). While spot cortisol levels in blood, saliva, and urine do not correlate with body mass index (BMI), a 9.8% increment in hair cortisol was associated with a higher BMI by 2.5 kg/m^2^ (119). Higher levels of cortisol per 1 cm of scalp hair are found in patients with endogenous CS at 679 (range 279-2500) ng/g (n = 6) when compared to HCs of 116 (range 26-204) ng/g (n = 32) (120). Hair cortisol has also been used to assess the adequacy of GC replacement in patients treated with hydrocortisone for AI (6). Thus, it could have potential for diagnosis or for follow-up assessment of cyclical CS where a longer term view of GC status may be helpful (121-124); however, it remains to be validated outside a research setting and should be considered in light of confounding factors such as sex, ethnicity, physical activity, light exposure, and medications used (125).

## Assessing GC Status: Tissue-specific GC Concentrations

Cortisol action in tissues is regulated by the enzyme 11β-HSD and the GC receptor (GR; the gene name is nuclear receptor subfamily 3, group C, member 1; NR3C1).

### Role of 11β-HSD

11β-HSD is a bidirectional enzyme that interconverts active cortisol to inactive cortisone (126). Cortisone is not synthesized in the adrenal gland. However, cortisone is produced in the kidney and gut and is largely dependent on the activity of 11β-HSD2, which inactivates cortisol to cortisone (127, 128). In metabolic tissues including liver, skeletal muscle, and adipose tissue, the reductase action of 11β-HSD type 1 (11β-HSD1) reactivates cortisol from cortisone, releasing ∼900 pmol of cortisol per 100 g of liver tissue per minute and ∼15 pmol of cortisol per 100 g of adipose tissue per minute, contributing to 30% to 40% of daily cortisol production (127, 128). The prereceptor interconversion between bioactive cortisol and inactive cortisone regulates the concentration of GC available for binding to the GR (126) and alters feedback onto the HPA axis (129).

Overexpression of adipose *HSD11B* in mice recapitulates features of metabolic syndrome without changes in circulating GC levels (130). Conversely, knockout of *HSD11B* in mice improves glucose tolerance, reduces weight, and improves the lipid profile also without affecting circulating cortisol levels (131). Although 11β-HSD1 enzyme activity in adipose tissue has been associated with insulin resistance and leptin levels (132, 133), *HSD11B* mRNA expression in subcutaneous or omental adipose biopsies was not associated with obesity (134). Genetic variants in the *HSD11B* gene have been associated with type 2 diabetes (135, 136) but not with obesity (135). Patients with CS with a functional deficit in *HSD11B* are protected from the adverse effects of GC excess (137).

Clinical trials using nonselective 11β-HSD inhibition have shown improved insulin sensitivity in patients with type 2 diabetes (138). A selective 11β-HSD1 inhibitor, ICNB13739, administered to patients with type 2 diabetes significantly lowered body weight and plasma glucose levels (139). In a recent proof-of-concept study in 32 men, a potent competitive 11β-HSD1 inhibitor, AZD4017, mitigated the metabolic side effects of prednisolone treatment by preventing worsening of hepatic insulin sensitivity, nocturnal blood pressure elevation, lipid metabolism, and bone turnover (preventing the fall in osteocalcin and N-terminal propeptide of type I collagen with prednisolone) while maintaining its desired anti-inflammatory action (assessed using the OX40 assay) (140).

These demonstrate that variation in 11β-HSD1 activity could alter GC action without necessarily being reflected in circulating GC levels. Thus, manipulation of 11β-HSD1 offers an attractive prospect to modulate GC action; quantifying the extent to which local cortisol is lowered in different tissues for therapeutic benefit while avoiding insufficient GC action could benefit from novel markers reflecting GC action (141).

### Variability in the Response of GC Receptor

**Figure 2.**
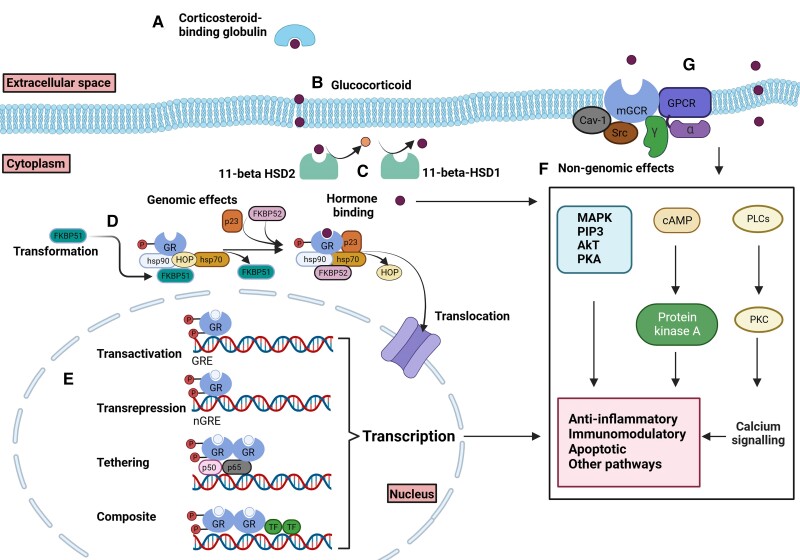
GC signaling pathway. (A) GCs are bound and transported by plasma protein. CBG is the main GC-binding protein that binds to 80% to 90% of the GCs. (B) GCs freely diffuse through cell membranes due to their lipophilic nature. (C) Cortisol is metabolized to inactive cortisone by intracellular enzyme, 11β-HSD type 2, which is expressed in abundance in some tissues (eg, distal nephron) (126), whereas 11β-HSD type 1, which is highly expressed in the liver, adipose tissue, and skeletal muscles, catalyzes conversion of cortisone to cortisol (134) (D) GC receptors reside predominantly in the cytoplasm of cells as part of a large multiprotein complex consisting of hsp20, hsp70, p23 (147). (D) Inside the cell, GR first interacts with hsp70 and HOP (147). HOP recruits GR:hsp70 to hsp90 and co-chaperone (including FKBP51 and GDKP52) bind to hsp90 as HOP is released. p23 binds and stabilizes the GR:hsp90 heterocomplex (147). FKBP51 inhibits nuclear transactivation of GR while FKBP52 may promote translocation (147). (E) GC-activated GR regulates gene expression via direct interaction to 1-GRE or 2-nGRE, 3- indirect interaction by tethering to DNA-bound transcription factors, or 4- composite binding to DNA while interacting with neighboring DNA-bound transcription factors. (F) Depending on the cell type, GC can also signal in a nongenomic manner through activating adenylate cyclase/PKA or PLC/PKC or the PIP3/Akt pathway resulting in changes in calcium concentration. (G) GCs can also have direct interaction with cell membranes and trigger a cascade of activity of kinases; however, this mechanism is less well described, or GCs can exert its action via binding to membrane GR, and phosphorylation of Cav-1 and PKB/Akt in a Src-dependent manner can lead to downstream signaling pathways.

Abbreviations: Cav-1, caveolin-1; CBG, corticosteroid-binding globulin; GC, glucocorticoid; GR, glucocorticoid receptor; GRE, glucocorticoid-responsive elements; GPCR, G-protein coupled receptor; 11β-HSD, 11beta-hydroxysteroid dehydrogenase; HOP, hsp-hsp90 organizing protein; MAPK, mitogen-activated protein kinase; mGR, membrane-bound GC receptor; nGRE, negative glucocorticoid-responsive element; PIP3, phosphatidylinositol (3,4,5)-trisphosphate; PKA, protein kinase A; PKB, protein kinase B; PKC, protein kinase C; PLC, phospholipase C; Src, steroid receptor coactivator.

The GR is comprised of 777 amino acids and is expressed in almost all human tissues and organs (142). Alternative splicing of NR3C1 precursor mRNA yields several GR splice elements that modulate GR function and affinity to GC, with the most abundant splice elements being GRα and GRβ (143). GC predominantly binds to GRα, whereas GRβ, being constitutively nuclear, can represses GRα-induced activity (143). Prior to ligand binding, GR predominantly resides in the cytoplasm and exists as a complex bound to 2 molecules of heat shock protein 90 (HSP90) and several other proteins (143). Upon binding to GC, GR undergoes conformational change, dissociates from HSP90, and is translocated to the nucleus (144) (Fig. 2). The ligand-bound GR dynamically interacts with GC responsive elements (GRE) or GRE half-sites in the promoter region of target genes or indirectly with other transcription factors to exert genomic effects (145). Variability in the GR response can therefore be influenced by GR expression level, the ligand-binding affinity of GR, posttranslational changes, the type of GREs, and the level of interaction with various coactivators or repressors (146).

Polymorphisms in the GR gene result in impaired function of GR as a transcriptional activator or repressor. Indeed, there is interindividual variation suggesting that there is variation in the set-point of the HPA axis. Genetic polymorphisms, such as N363S (rs6195), are associated with increased GC action, increased BMI, and reduced bone mineral density (148). Similarly, individuals with BclI polymorphism (rs41423247) also exhibit increased GC sensitivity, characterized by increased central obesity, insulin resistance, hypertension, and depression (149). By contrast, carriers of ER22/23EK (rs6189 and rs6190) polymorphism had reduced GC action and lower BMI and cardiovascular risk (149). Individuals who were homozygous in the intronic region of *PDGFD* locus (rs591118) are more likely to develop adrenal suppression following treatment with GC compared to those who are homozygous wild-type (150). Thus, variability in the action of the GR can affect the GC action beyond alterations in circulating GC levels, and in vivo and in vitro assessment of GC sensitivity has been proposed (6).

### Body Weight/Appetite

Changes in body weight can reflect alterations in GC action. GC deficiency is associated with weight loss due to anorexia as well as salt and water depletion (64). Adrenalectomy in rodents is associated with decreased food intake, which is reversed with GC replacement, mediated through an effect on appetite-controlling neuropeptides in the hypothalamus, including insulin (151), oxytocin, and corticotrophin-releasing hormone (152). Additionally, patients with PAI have delayed gastric emptying, which may further contribute to anorexia and weight loss (153).

By contrast, GC excess may increase oral intake and cause weight gain (154). In vitro and in vivo studies also showed that GCs can increase hypothalamic endocannabinoids, which have been shown to increase hypothalamic AMP-activated protein kinase (AMPK) activity resulting in increased appetite (13, 155). In a randomized controlled trial of pulsed high-dose dexamethasone vs standard prednisolone therapy, 17 of 40 (42.5%) reported some increase in appetite and 19 of 40 (47.5%) gained at least 1 kg of body weight in both groups over 24 weeks (156). In healthy men, energy intake was increased compared to baseline intake after 4 days in those receiving methylprednisolone (40 mg per day, n = 10) by 4554 kcal per day compared to 2867 kcal per day in those receiving placebo (n = 10) (157).

In a bidirectional Mendelian randomization study, single nucleotide variants independently associated with morning plasma cortisol were obtained from the CORtisol NETwork consortium. Of note, the CORtisol NETwork consortium undertook genome-wide association studies for plasma cortisol and identified that <1% of plasma cortisol variance is accounted for by variation in chromosome 14. The locus identified spans SERPINA6 encoding cortisol-binding globulin and SERPINA1 encoding α1-antitrypsin (which inhibits cleavage of the reactive center loop that releases cortisol from CBG) (158). Surprisingly, genetically predicted cortisol levels were negatively associated with BMI class and vice versa, with genetically predicted blunted morning cortisol secretion being associated with obesity (159). Given that the morning cortisol rise is an important feature of the physiological HPA axis, this suggests that disruption to the circadian rhythm of cortisol secretion could contribute to the development of obesity (160).

### Blood Pressure

Changes in blood pressure occur in both AI (including SAI where mineralocorticoid production is preserved) (161) and GC excess (3). In patients with PAI, 88% have hypotension and 12% have symptomatic postural dizziness, although mineralocorticoid deficiency leading to salt and water loss is a dominant factor in PAI (162). GCs also contribute to blood pressure via the transcriptional expression of the enzyme phenylethanolamine-N-methyltransferase, which is responsible for the conversion of norepinephrine to epinephrine in the adrenal medulla (163). GCs also contribute to catecholamine-induced vasoconstriction (164). Therefore, GC deficiency results in a relative decrease in epinephrine (reduced vasoconstriction and systolic blood pressure) and an increase in norepinephrine (increased heart rate) (165).

Hypertension is observed in 80% of patients with CS (165) through a multifactorial mechanism, including a reduction in vasodilation via inhibition of nitric oxide synthase expression (166) and modulation of catecholamine synthesis (165). Cortisol and aldosterone have the same affinity for the mineralocorticoid receptor, but 11β-HSD2 inactivates cortisol under basal conditions. However, the enzyme can become saturated at high cortisol levels, eg, in ectopic-ACTH CS, resulting in excess mineralocorticoid action and hypertension.

### GC Status and Muscle Function

Up to 50% of patients with PAI experience generalized weakness, muscle cramps, and fatigue (167, 168), whereas around 40% to 60% of patients with CS present with muscle weakness (169-171). Mechanisms contributing to muscle weakness in GC deficiency include impaired muscle carbohydrate metabolism, depleted muscle glycogen stores, and suppression of adrenaline-induced activation of hepatic and skeletal muscle glycogen phosphorylase (168). By contrast, GC excess induces myopathy via activation of major cellular proteolytic systems [eg, ubiquitin-proteasome system, lysosomal system (cathepsins), and calcium-dependent system (calpains)], inhibition of protein synthesis (172), alteration of growth factors (eg, IGF-1, myostatin), and testosterone levels (172). Patients with CS tend to have predominant postural and proximal muscle weakness due to preferential atrophy of fast-twitch glycolytic (type IIb) muscle fibers (173, 174).

### Biomarkers for the Assessment of Muscle Status

GC-induced muscle atrophy is associated with changes in the muscle secretome. SerpinA3N is a secretory serine protease inhibitor that has a wide range of effects including suppression of osteoblasts, as well as being a myokine (secreted into the blood in response to muscle contraction) that is released in association with muscle atrophy. Expression of skeletal muscle *SerpinA3N* is increased in vitro from skeletal muscle (175); circulating levels of serpina3n were increased by 2.5-fold in 9 patients with CS (175), suggesting that it could represent a potential biomarker of GC-induced muscle atrophy pending larger studies (175).

Myostatin is also a protein produced in muscle tissue that negatively regulates muscle mass. Myostatin administration induced a 35% to 50% decrease in muscle mass (176), whereas an antimyostatin antibody attenuated chronic kidney disease associated muscle proteolysis in murine models (177). In in vitro studies, dexamethasone treatment increased myostatin mRNA levels at 24 hours, but the rise in myostatin protein levels was only observed at 36 hours (178). This discrepancy in mRNA and protein levels suggests that myostatin may be upregulated transiently at the beginning of GC exposure, but it may not be the main pathway that mediates the protein catabolic process (178). In animal studies, daily intraperitoneal administration of dexamethasone at 60, 600, and 1200 μg/kg body weight for 5 days resulted in a dose-dependent reduction in weight and muscle atrophy, and this decrease was associated with a marked upregulation of myostatin mRNA by 66%, 450%, and 527.6% compared to pair-fed control rats (179). However, the expression of myostatin mRNA and protein levels was not sustained, and levels returned to basal levels when dexamethasone treatment was extended to 10 days (179). Another research group reported similar findings in that in vivo myostatin expression was time-dependent with a transient elevation at day 5 during the 10-day dexamethasone treatment (180). A case-control study showed that serum levels of myostatin did not significantly differ in 30 patients with CS from HCs (181). Inder et al also showed that short-term oral dexamethasone treatment (4 mg for 4 days) induced relative changes in plasma testosterone (male: 15.0 vs 11.3 nmol/L; female: 1.8 vs 0.5 nmol/L) but skeletal muscle androgen receptor expression and skeletal muscle myostatin mRNA levels were unchanged (182). Overall, these studies suggest that muscle loss associated with GC is mediated in part by transient upregulation of myostatin at the beginning of GC exposure, but this may not be the main pathway mediating GC-induced muscle atrophy.

GC can also cause muscle atrophy by altering the production of growth factors (eg, IGF-1) that control muscle mass development. IGF-1 stimulates protein synthesis and differentiation of muscle satellite cells (183) and downregulates the muscle proteolytic systems by inhibiting the expression of atrogenes that cause muscle atrophy, such as atrogin-1, MuRF-1, and cathepsin L, which are induced by GC (184, 185). Muscle IGF-1 overexpression prevents muscle atrophy in GC-treated animal models. However, although IGF-1 appears to have a dominant effect in reversing GC-induced muscle atrophy, systemic administration of IGF-1 could be limited by its hypoglycaemic (186) and cardiac hypertrophic actions (187). In human studies, short-term administration of GC increases serum IGF-1 levels (182, 188, 189), but levels of IGF-1 in tissue fluid remain unchanged (189). Skeletal muscle expression of IGF1 mRNA was significantly downregulated, suggesting a distinct tissue-specific effect on IGF1 action that could be partly mediated by GC-induced changes in plasma testosterone levels (182). Therefore, although IGF-1 appears to have a beneficial effect in reversing GC-induced muscle catabolism, further studies are needed to determine its independent effect.

### Imaging in Assessment of Muscle Status

Dual-energy X-ray absorptiometry and bioelectrical impedance analysis can be used in the assessment of muscle mass, but due to the nonuniform nature of muscle atrophy in CS (190) and the reliance on a constant for hydration of fat free mass (191), dual-energy X-ray absorptiometry and bioelectrical impedance analysis could give inaccurate results.

Computed tomography (CT) and magnetic resonance imaging are considered gold standards for assessing muscle density and muscle composition as both modalities quantify intramuscular fat content based on attenuation characteristics to discern between fat and muscle tissue (192). Muscle density has been shown to better reflect handgrip strength and functional outcomes compared to muscle mass (193). In patients with CS, psoas muscle density measured by CT negatively correlated with urinary free cortisol (*r* = −0.30) (194), whereas treatment of GC excess increased CT-measured cross-sectional area of thigh and calf muscle (195). The use of CT or magnetic resonance imaging is limited in practice due to cost, radiation, and operational complexity. Thus, ultrasound has emerged as a reliable alternative to measure muscle mass (ie, the thickness) and structure (ie, the echo density), which comprise muscle composition (196). Muscle thickness was similar, but ultrasound-determined echo intensity of vastus lateralis, tibialis anterior, and medial gastrocnemius were significantly higher in patients with active CS compared to those in remission, indicating that muscle structure recovery precedes muscle mass recovery following resolution of hypercortisolemia (196). Needle biopsy and surface electromyography showed atrophy of type II fibers and slowing of conduction in patients with CS (197). Atrophy of muscles reduce muscle fiber length and cause architectural changes that contribute to reduced force production capacity (197). Slowing of muscle fiber conduction velocity could be related to the suppressive effect of GC on sacrolemmal excitability. The preferential GC impairment of fast fibers translates to a greater difference in muscle fiber contraction velocity of vastus lateralis and vastus medialis muscles (∼26%) compared to that of tibialis anterior muscle (∼11%) between patients with CS and age-sex matched HCs (197).

However, these changes are also observed in other conditions such as aging and neuromuscular diseases and thus lack specificity for the diagnosis of CS (198, 199). Thus, it is possible that imaging can be used as an adjunct to provide objective evidence for changes in GC status; however, specificity may be lacking for diagnosis. Although emerging technologies provide a means of assessment of muscle quality changes, there is currently no standardized imaging modality that is preferred for use as a diagnostic tool for assessing changes in muscle mass.

### Immunomodulatory Effects of GC

GCs have a broad range of immuno-modulatory and immune-suppressive actions on both innate and adaptive immunity, including inhibiting lymphocyte activation, promoting lymphocyte and dendritic cell apoptosis, and downregulation of inflammatory cytokines. It is worth noting that many studies have used much higher doses of GCs than are used clinically for GC replacement (152, 200).

In patients with PAI, population studies have demonstrated an increased risk of infection, which contributes to an increased mortality rate (201, 202). This may be explained by impaired NK-induced cellular toxicity. NK cells in patients with PAI have a reduced expression of the activating receptors NKG2D and NK-46 (186), which may explain the propensity for increased severe bacterial infections. Similarly, peripheral blood mononuclear cells (PBMC) from patients with PAI have a deficient chemokine response to interferons reflective of the adaptive immunity and the ability to defend against viral infections (203).

Whether these findings are secondary to imperfect GC replacement therapy or a feature of AI is difficult to tease apart. One study showed a normalization of immune cells implicated in innate bacterial defense and viral immunity in response to changing to a modified-release hydrocortisone preparation, linking this to a reduction in viral illnesses (101). However, it is not clear how a mild excess of GC replacement could affect immune cell function (204). Further work is needed to assess the effects of different replacement regimens on different immune cell populations.

In CS, there are a variety of changes within the innate and adaptive immune system that increase the risk of severe and opportunistic infections and contribute to a low-grade inflammatory state (205). These include an increase in neutrophils [due to increased release of polymorphonuclear leucocytes (PMNs) from the bone marrow and reduced apoptosis and extravasation of PMNs leading to an increased PMN half-life in the blood stream]. GC excess also reduces NK cell activity and macrophage phagocytic activity due to a reduction in circulating monocytes. Adaptive changes include a relative reduction in CD4+ T cells, a relative increase in CD8+ T cells, and a reduced B lymphocyte count (190), increasing susceptibility to intracellular and opportunistic infections.

Increased proinflammatory adipokines, altered tumor necrosis factor α regulation, IL-6, and C-reactive protein also contribute to the chronic, inflammatory state that is thought to contribute to the pathogenesis of the other clinical complications, including diabetes, atherosclerosis, and osteoporosis, with an increased risk of cardiovascular disease that persists after remission (206).

It has also been observed that CS patients experience rebound autoimmune diseases during remission. This may be due to overactivity of previously suppressed immune function. The extensive effects of GCs on the immune system are beyond the remit of this review; further in-depth discussions on the immune system changes can be found in a recent comprehensive review (205). A better understanding of changes in the immune system could offer further biomarkers that could be used to assess GC status.

### GC Effects on Glycemia

The mechanisms by which GCs exert a diabetogenic effect are complex and are context dependent, but proposed effects include increased insulin resistance, both increased or decreased insulin secretion, and stimulation of hepatic gluconeogenesis. Additional interorgan signaling and tissue-specific effects in vivo further contribute and amplify GC's diabetogenic effects. The reader is directed to a recent comprehensive review focusing on glucocorticoid effects on glycemia (207).

Insulin resistance occurs both peripherally and in the liver. As a result of hepatic insulin resistance, there is removal of insulin-mediated repression of hepatic gluconeogenesis. Peripherally, GCs directly disrupt insulin signaling through the downregulation and phosphorylation of IRS1, PI3K, and PKB-AKT (208-210). This inhibits insulin-induced recruitment of the glucose transporter 4 in skeletal muscle, reducing insulin sensitivity and glucose uptake (211). Additional effects on protein degradation increase serum amino acids, providing substrates for gluconeogenesis (212, 213).

De novo glucose production predominantly occurs via hepatic gluconeogenesis via upregulation of the enzymes phosphoenolpyruvate carboxykinase and glucose-6-phosphatase. The action of GCs on other tissues, such as skeletal muscle (via protein catabolism) and lipolysis in white adipose tissue, increase gluconeogenic precursors that are shuttled to the liver (172, 214), further driving hepatic gluconeogenesis. Understanding the effects of GCs on insulin secretion in vivo is challenging due to the duration of GC exposure and compensatory effects that occur with increased exposure duration, which is difficult to elucidate in in vitro and ex vivo studies. Preclinical studies have demonstrated an increase in beta cell mass following GC exposure in a dose-dependent manner (215); however, this has not been demonstrated in humans where exposure to GC resulted in minimal changes to beta cell mass (216). In humans, an acute single oral GC dose has been shown to acutely impair the initial beta cell response to hyperglycemia with increased AUC_glucose_ and reduced AUC_c-peptide_, but this demonstrated acute recovery with subsequent increased insulin secretion (both fasting and in response to a mixed meal test) 24 hours later. In individuals exposed to 15 days of treatment, there was evidence of increased c-peptide secretion but increased levels of fasting and postprandial AUC_glucose_, suggesting that the increase in insulin is insufficient to maintain euglycemia (217).

In addition, studies have also demonstrated increased basal and stimulated glucagon levels in participants following GC administration (217, 218), further contributing to hyperglycemia. However, the literature is not consistent, with other studies failing to demonstrate this (219). This may be explained by differences in the insulin sensitivity in the individuals studied. It has been suggested that additional alpha cell disturbances may be present in those with reduced insulin sensitivity (219).

AMPK is a key regulatory enzyme of appetite, lipid, and carbohydrate metabolism and is believed to mediate some of the deleterious metabolic effects of excess GC (220). AMPK activity in visceral adipose tissue was inversely associated with 9:00 Am cortisol levels and urinary free cortisol (220). The effects of AMPK inhibition have demonstrated impaired insulin-mediated glucose uptake by skeletal muscle and adipose, increased hepatic glucose output, reduced beta-cell glucose-stimulated insulin secretion, and increased lipogenesis and fat storage (221). Activation of AMPK has been shown to reverse GC-induced hepatic steatosis and effects on glucose metabolism (222). In nondiabetic patients established on GC for inflammatory disease, treatment with metformin (an activator of AMPK) for 12 weeks reduced truncal subcutaneous fat (−3835 mm^2^), improved insulin resistance and glucose concentrations, and reduced inflammation and progression of carotid intima thickness (223), suggesting that concomitant use of metformin can mitigate the deleterious effects of GC excess (223).

In summary, there is evidence that, through a variety of mechanisms, GC deficiency may result in hypoglycemia, especially in children (66), whereas chronic GC excess can result in hyperinsulinemia and, in those at risk, hyperglycemia (3).

### GC Effects on Lipid Profiles

The effects of GC on lipid dysregulation are multifactorial and involve both direct and indirect pathways. GC promotes lipolysis of mature adipocytes (214, 224) but stimulates lipogenesis on preadipocyte cells (224). Low to moderate concentrations of GC (1-10 µM) stimulate lipolysis of mouse adipocyte 3T3-L1 cells, but high doses of GC suppress lipolytic rate (224). Lipolysis is likely to be mediated via increased intracellular cAMP levels and protein kinase A activity, and this effect could be blunted by downregulation of cyclic-nucleotide phosphodiesterase 3B, a major enzyme responsible for cAMP hydrolysis (214, 224). GC-induced lipolysis may also in part be mediated via a direct upregulation of GR target such as angiopoietin-like 4, an adipokine that inhibits lipoprotein lipase activity that is involved in lipolysis (225). In vivo studies reported a 60% rise in fatty acid palmitate level when circulating cortisol concentrations increased from 245 ± 12 nmol/L to 888 ± 7 nmol/L during a 6-hour hydrocortisone infusion (2 μg/kg/min) in the presence of a pancreatic pituitary infusion clamp (226).

Notably, in an individual, prevailing levels of insulin, somatostatin, and adrenaline concentrations could modulate the lipolytic effects of GC. Healthy volunteers (n = 15) who underwent a paired 2-step hyperinsulinaemic euglycemic clamp with a concurrent overnight HC (0.2 mg/kg/h) or saline infusion (at least 2 weeks apart, in random order) along with adipose tissue microdialysis had lower circulating free fatty acid levels during the high insulin concentration phase compared to low insulin level period compared to placebo (low insulin with HC vs placebo: 564 ± 40 vs 403 ± 38, *P* < .01 μmol/L·h; high insulin with HC vs placebo: 712 ± 35 vs 443 ± 38 μmol/L·h, *P* < .005) (227). During the clamp studies, glucose infusion rates decreased in response to insulin infusion in the HC group compared to placebo, suggesting a reduced glucose disposal rate, representing an increase in systemic insulin resistance. Endogenous glucose production did not differ between the HC or saline group, but the glucose production rate in the presence of a high insulin level was lower in the HC arm compared to saline, suggesting hepatic insulin resistance (227). Insulin also suppresses the magnitude of GC-induced lipolysis potentiated by adrenaline and stimulates lipoprotein lipase activity (226). As a result, despite a possible increase in lipolysis, an overall increase in lipogenesis (224) and hepatic and systemic insulin resistance occurs (224) and, in turn, promotes hepatic gluconeogenesis (via induction of glucose-6-phosphatase and phosphoenolpyruvate carboxykinase) (228). Adipogenesis promotes free fatty acid release into the circulation and ectopic storage of lipids in liver and skeletal muscle (229). Effects of GC-induced adipogenesis are more notably observed in the visceral adipose tissue, which has a relatively higher number of GC receptors (230). Consequently, expansion of visceral adipose tissue and reduction of subcutaneous adipose tissue further impair liver and muscle sensitivity, leading to cardiometabolic disease (231). In summary, GC hypercortisolism promotes lipolysis but hyperinsulinemia above a certain threshold suppresses GC lipolysis and lipogenesis and insulin resistance predominates (232).

Dsylipidemia is a well-recognized feature in patients with hypercortisolism. Patients with active CS exhibit increased very low density lipoprotein, low-density lipoprotein, and triglycerides, but no change in the lipid profile was observed on remission of CS (233). GC replacement in patients with SAI reduced plasma cholesteryl ester transfer protein activity, resulting in increased high-density lipoprotein (HDL) cholesterol levels (234) and HDL size (235), but triglyceride or low-density lipoprotein cholesterol levels were unchanged (234). Consistent with this, daily administration of low-dose dexamethasone to healthy people for 3 weeks increased HDL cholesterol (236). By contrast, a large study of 2424 patients with hypopituitarism showed no significant changes in lipid levels between patients treated with GC replacement and those who were GC sufficient (237). Further clinical data are needed prior to consideration of lipid as a potential marker of GC status.

### Adiponectin and Leptin

In humans, treatment with 0.5 mg of dexamethasone 4 times daily for 2 to 5 days reduced insulin sensitivity, but plasma levels of adiponectin, resistin, and leptin remained unchanged (238). Administration of hydrocortisone (25 mg) reduced adiponectin levels at 30 and 60 minutes both in patients with and without obesity (239). Adiponectin levels are lower in nonobese patients with CS (20.9 mcg/mL) than in HCs (30.9 mcg/mL), but levels of adiponectin did not differ between patients with CS and obesity compared to those with obesity alone (20.1-22.1 mcg/mL) (239). Thus, adiponectin could be useful in identifying GC excess but only in the absence of obesity.

Leptin levels were increased dose-dependently after 1 day of a 4-day course of oral hydrocortisone (at either 40 or 160 mg daily) by more than double after the higher dose in women; however, levels returned to baseline level by the fourth day and were less increased in men (240). Diurnal secretion of leptin is preserved in patients with CS (241). In patients without obesity, fasting leptin levels are 2-fold higher in patients with CS compared to nonobese controls (33.5 vs 14.2 ng/mL) (241). In contrast, a smaller difference in leptin levels was observed between patients with obesity compared to BMI-matched HCs (55.0 vs 41.7 ng/mL) (241). Leptin levels remained stable during the immediate postoperative period (10 days), despite a concurrent decrease in cortisol and ACTH levels after surgical cure of CS (241, 242). This lack of change in leptin levels after successful surgery could be more associated with the lack of change in BMI and adiposity in the immediate postoperative period (241) as others have reported a reduction in leptin concentration and BMI in patients with CS 3 to 6 months after surgery (243). In summary, hypercortisolaemia may indirectly induce body fat changes and hyperinsulinemia, which in turn could influence leptin concentrations (241, 243). Overall, there are several markers that may be used to assess GC status; however, given the tissue-specific responsiveness to GC that exists (244), there is no single marker that accurately reflects GC status in isolation.

## Novel Markers That Could Be Used in the Future to Quantify GC Status

Please see Table 4.

**Table 4.** Summary of novel markers in the assessment of GC status

Novel marker	Associated changes and use to assess GC status
Urinary steroid metabolome	Limited data Use of multisteroid profiling using tandem mass spectrometry in the evaluation of patients with adrenal tumors Stepwise change in the pattern of 24-hour urinary cortisol excretion seen in NFAT to MACS to CS (246) Use in AI and GC replacement with a shift toward healthy controls in patients taking dual-release hydrocortisone (247)
Genetic markers	
Micro RNAs	
*miR-122-5p*	Reduction in PBMC miR-122-5p on GC exposure (248)
*miR-182-5p*	Upregulated in Cushing's disease compared with controls (249)
*miR-96-5p*	Upregulated following dexamethasone administration (249)
*miR-185-5p*	Downregulated following dexamethasone administration (249)
GDF15	GCs repress transcription of GDF15 mRNA in vivo (250) Evaluated in patients with PAI. Patients with PAI had higher circulating GDF15 levels, which decreased following HC infusion Evidence of dose-dependent decrease in GDF15 levels following low-, medium-, or high-dose oral GC replacement (251)
GC-responsive elements TSP1	Matricellular protein expressed in endothelial cells, monocytes, macrophages, and adipocytes (252, 253) Increased *TSP1* mRNA in osteoblast, endometrial, and PBMCs following dexamethasone administration (254, 255) Increased levels in patients with CS compared with healthy controls (254) Increased levels in patients with AI following increase in hydrocortisone replacement therapy dose (254) Potential for use with osteocalcin to identify CS (256)
Others to be further evaluated	
Lysozyme C High-mobility group protein 2 Nucleophosmin-1	These have been identified through proteomic analysis of PBMC secretome from healthy volunteers exposed to dexamethasone (255)
FKBP5	Potential biomarker of cortisol activity at the GR (257, 258) Reduces GR activity through reduced cortisol binding affinity (259) Increased levels following infusion of hydrocortisone and dose of prednisone (260, 261) Increased levels in ACTH-dependent CS compared with controls (with normalization following curative surgery) (257) Reduced levels with GC antagonist (261)
FKBP5 methylation	Negative correlation with basal serum cortisol levels and 24 hours following ACTH stimulation (262)
REDD1	Increased following GC administration—limited to preclinical models only (263) Molecular target for GCs mediating skin and muscle atrophy (264, 265)
Bone turnover markers	
Osteocalcin	Reduced osteocalcin following GC administration (266-268) Negatively correlated with serum cortisol levels in patients with active CS (269), with increased levels following transsphenoidal or adrenal surgery for CS (270) No studies evaluating individuals with milder degrees of cortisol excess
Total and bone-specific ALP PINP	Less sensitive to GC effects with milder and delayed decrease following GC administration (268)
SerpinA3N (secretory serine protease inhibitor)	Released in association with muscle atrophy Increased in CS (173)

Abbreviations: AI, adrenal insufficiency; ALP, alkaline phosphatase; CS, Cushing’s syndrome; GC, glucocorticoid; GR, glucocorticoid receptor; MACS, mild autonomous cortisol secretion; NFAT, nonfunctioning adrenal tumor; PAI, primary adrenal insufficiency; PBMC, peripheral blood mononuclear cell; PINP, procollagen type I carboxy-propeptide; REDD1, regulated in development and DNA damage response 1.

### Metabolomic Assessment

To date, there have been relatively few studies evaluating whether there is a metabolomic signature reflective of healthy GC status and how it may be altered after a changed GC status. Recently, Kachroo et al used metabolomic assessment to identify 17 steroid metabolites that were altered in patients treated with inhaled corticosteroid for asthma (245). They highlight that genetic variation can underpin the heterogeneity in treatment response and adrenal suppression with inhaled steroids (245). A significant proportion of patients on inhaled steroids were found to have low cortisol levels, although they may yet have increased GC status due to inhaled steroid use, highlighting a clinical example where GC levels may not reflect GC status (245).

Patients with adrenal tumous, including those with nonfunctionating adrenal tumors or those with mild autonomous cortisol secretion (MACS) or adrenal CS, were assessed by multisteroid profiling of 24-hour urine by tandem mass spectrometry (271). Those with MACS or adrenal CS had a decrease in 24-hour urinary excretion of androgen metabolites (androsterone, etiocholanolone, dehydroepiandrosterone) and of pregnenetriol (metabolite of 17-hydroxypregnenolone) and an increase in the 24-hour urinary excretion of cortisol and tetrahydro-11-deoxycortisol (metabolite of 11-deoxycortisol) (271). Notably, there was a step-wise change in the pattern of urinary steroids from those with nonfunctionating adrenal tumors to MACS to CS, suggesting that such an approach could be used to aid in the objective assessment of GC status (271). Similarly, in patients with AI (n = 50), dual-release hydrocortisone compared with thrice-daily hydrocortisone decreased cortisol exposure and shifted the urinary steroid metabolome toward that of HCs and thus could potentially be used as a tool to optimize GC replacement (247).

### Growth Differentiating Factor 15

Growth differentiating factor 15 (GDF15) (previously called MIC-1) is a member of the transforming growth factor β family, expressed by cells in response to stress (250, 251). As a marker of stress, it can be elevated in both physiological and pathological states, including pregnancy, exercise, chronic inflammatory disease, renal failure, and cardiac failure (250). In vivo, GCs repress transcription of GDF15 mRNA, suggesting that GDF15 could potentially be used as a downstream marker of GC signaling in the endoplasmic reticulum stress pathway (272).

Utility of GDF15 has been explored in patients with PAI (251). In a proof-of-concept study, patients with PAI (n = 10) without GC replacement for 39 hours (cortisol levels: PAI 47 ± 17 nmol/L, controls 371 ± 72 nmol/L) had higher circulating GDF15 levels (PAI: 739 ± 226 pg/mL) compared to HCs (498 ± 168 pg/mL) (251). However, these elevated levels in patients with PAI were reduced to 559 ± 120 pg/mL after a 22-hour hydrocortisone infusion (251). Additionally, there was a dose-dependent decrease in GDF15 levels (mean ± SD) in patients on low- (491.0 ± 157.7 pg/mL), medium- (427.0 ± 152.1 pg/mL), or high- (360 ± 143.1 pg/mL) dose oral GC replacement (251). GDF15 levels were also significantly lower in patients with congenital adrenal hyperplasia after a 300-minute hydrocortisone infusion (hydrocortisone 547.0 pg/mL vs placebo 606.0 pg/mL) (251). Likewise, a 22-hour hydrocortisone infusion (0.008-0.024 mg/kg/hr) reduced GDF15 levels in a dose-dependent manner in patients with PAI (baseline 754 vs 610 pg/mL at 22 hours) (251). Although this is early data, GDF15 could have a potential utility to contribute as a marker of insufficient GC status pending further study in larger cohorts (251).

### **MicroRNAs**—**miR-122-5p**

MicroRNAs (miRNAs) are a group of small noncoding RNAs that regulate gene expression. Given their presence in many extracellular compartments such as plasma, serum, urine, and saliva, miRNAs hold potential as noninvasive biomarkers (273). In particular, miR-122-5p has been identified as having a key role in regulating gene expression in several cancers, including breast cancer and pancreatic ductal adenocarcinoma (274). GC exposure was associated with a 2-fold reduction in levels of miR-122-5p in circulating peripheral blood mononuclear cells (*P* = .009) (248). By contrast, circulating miR-182-5p was upregulated in Cushing’s disease in comparison to controls, with an area under the receiver operating characteristic (AuROC) curve of 0.89 (249). After 1 mg dexamethasone treatment, miR-96-5p was upregulated and miR-185-5p was downregulated (249) in postdexamethasone samples. Thus, while currently only used in the sphere of investigative research, miRNA biomarkers could in the future aid in the assessment of GC status.

### GREs

A given GC dose can have variable efficacy and side-effect profile in different individuals. At present, no clinically useful biomarker of GC activity has been established for diagnosis or for use in therapeutic monitoring.

Thrombospondin-1 (TSP1) is a matricellular protein that is widely expressed in endothelial cells, monocytes, macrophages, and adipocytes (252, 253). It has a plasma half-life of 9 hours and is unaffected by age, sex, BMI, or commonly administered medications for hypertension, hyperlipidemia, or diabetes (275, 276). *TSP1* mRNA was increased by dexamethasone in different cell types (eg, osteoblast, endometrial, or peripheral blood mononuclear cells) in vitro (277), and the antiangiogenic effect of cortisol on endometrial endothelial cells was abolished by small interfering RNA against *TSP1* mRNA, indicating that TSP1 may be a downstream GC effector (278). A single 4-mg dose of dexamethasone increased plasma *TSP1* mRNA in peripheral blood mononuclear cells in healthy individuals at 12 hours (254). Median (interquartile range) morning plasma TSP1 was significantly higher in patients with CS (n = 8) compared to healthy volunteers (n = 20) [CS: 638 (535-756) ng/mL vs HCs: 272 (237-336) ng/mL] (254). A threshold of 400 ng/mL identified CS with 100% sensitivity and 85% specificity (AuROC 0.94) (254). Likewise, morning plasma TSP1 levels were increased in patients with AI (n = 16) when the maintenance dose of HC was increased from 20 to 30 mg within a week from a baseline level of 139 (86-199) ng/mL to 256 (133-516 ng/mL) (254). These studies suggest that TSP1 is a GC-responsive protein in humans that could be used to reflect GC action. To improve diagnostic sensitivity and specificity, the ratio of TSP1 to osteocalcin taken from a morning blood sample was used in a study of 20 healthy volunteers and 19 people with CS (256). A TSP1:osteocalcin ratio of >73 reliably identified individuals with CS with an AuROC of 0.997 (256), highlighting it as a promising potential biomarker of GC status. Proteomic analysis of the secretome from PBMC cells isolated from healthy volunteers exposed to 100 ng/mL of dexamethasone yielded 3 additional novel candidate GC-responsive proteins that altered with GC exposure (eg, lysozyme C, high-mobility group protein 2, and nucleophosmin-1), which could be further explored in future studies (255).

### FKBP5 Expression

FKBP5, a protein encoded by the *FKBP5* gene, is a potential biomarker of cortisol activity at the GR (257, 258). FKBP51 is a cochaperone of HSP90, which together form part of the GR chaperone complex (279). When bound to the complex, FKBP51 decreases GR activity by reducing its cortisol-binding affinity and preventing its translocation to the nucleus (259). Thus, FKBP51 interacts with the GR to maintain it in an unbound inactive state (259). Upon GC binding to the GR, FKBP51 dissociates from the complex and is replaced by FKBP52 enabling nuclear translocation of GR (258, 279). Once migrated into the nucleus, the GR binds to GRE to affect gene expression, including of *FKBP5* (258, 279). This leads to an increase in the amount of *FKBP5* mRNA and FKBP51 protein, which in turn restricts GR nuclear translocation, thus completing an intracellular negative feedback loop for GR activity (258, 279). Thus, FKBP5 could be used as a biomarker to reflect GC receptor action.

FKBP5 expression levels positively correlate with serum cortisol (*r* = 0.419) in patients with PAI (260). Furthermore, an infusion of 100 mg hydrocortisone in patients with PAI off medications induced a 1.35-fold increase in FKBP5 (260). However, at very low cortisol levels, no correlation was found between *FKBP5* expression and serum cortisol levels, although it negatively correlated with plasma ACTH (*r* = −0.399) (260).

Oral administration of a single dose of 25 mg of the synthetic GC prednisone increased FKBP5 mRNA expression by 16-fold within 4 hours, and levels returned to baseline within 24 hours of prednisone discontinuation (261). A single dose of mifepristone was equally effective in reducing prednisone-induced FKBP5 mRNA levels back to unstimulated levels within 4 hours (261). Likewise, repeated administration of the GC antagonist once daily for 14 days completely ablated prednisolone-induced FKBP5 (261).

Median (range) *FKBP5* mRNA levels were higher in patients with ACTH-dependent CS (n = 24) than in HCs [2357.3 (1071.45, 17215.80) vs 918.7 (661.68, 1735.91)] (257). FKBP5 mRNA strongly correlated with cortisol levels both before and after surgery (*r* = 0.72-0.85) (257). Notably, after successful surgical treatment of CS, *FKBP5* mRNA expression decreased to levels similar to those of HCs (CS postsurgery 1148.7 ± 372.8, HCs 918.7 ± 311.1) but remained elevated in patients with failed surgical treatment (2505.3 ± 1843.4) (257). Overall, the change in *FKBP5* mRNA expression with GC exposure shows promise as an in vivo transcriptional biomarker of GC receptor activity that could contribute to assessment of GC status.

### FKBP5 Methylation

Epigenetic changes in gene expression due to environmental influence cause alterations in gene activity rather than in gene sequence (280). The most extensively investigated epigenetic modification is DNA methylation (DNAm), which acts to repress transcription of the gene and can result in gene silencing via changes in chromatin structure (281).

Changes in chromatin structure involve intron 7 of the GREs, which is thought to contain a transcription enhancer (282). Less DNA methylation of GREs at intron 7 is associated with higher induction of FKBP5 by GC receptor activation and thus can result in differential expression and responsiveness of FKBP5 to GR (282).

Several studies have begun to examine the role of DNAm in relation to GC status, in particular, FKBP5 promoter methylation levels (262). In healthy volunteers, DNAm of the *FKBP5* gene in leukocytes was measured before and after ACTH stimulation (262). *FKBP5* methylation was negatively correlated with basal serum cortisol (*r* = −0.439) (262). The change in *FKBP5* methylation at 24 hours after ACTH negatively correlated with the increase in cortisol levels at 1 hour after ACTH (*r* = −0.537) (262).

Despite no significant changes in the *FKBP5* methylation status at 24 hours, in vitro studies have suggested changes in methylation of *FKBP5* following longer term GC exposure for up to 4 weeks (283, 284). In fact, patients with CS were found to have reduced *FKBP5* methylation of intron 7 compared to HCs, although there was no difference between cured vs active CS patients (cured CS 77.1%, active CS 73.7%, and HCs 79.7%) (285).

Several studies have explored the potential of *FKBP5* methylation as a biomarker of chronic GC load. *FKBP5* methylation levels after 4 weeks of corticosterone exposure in mice significantly correlated with average corticosterone load (284). Overall, this supports the notion that FKBP5 expression levels reflect short-term GC exposure, whereas FKBP5 methylation reflects longer term GC exposure.

### REDD1

Regulated in development and DNA damage response 1 (*REDD1*) is a hypoxia and stress-response gene that inhibits mammalian target of rapamycin complex-1 activity (286). It has a low level of expression in basal conditions and is induced in response to hypoxia, stress, DNA damage, or transcription factor activation (286).

Recent studies have identified the hypoxia and stress-response gene *REDD1* as a molecular target for GCs that mediates cutaneous and skeletal muscle atrophy in skin (264). In REDD1 knockout mice, skin and muscle atrophy are averted (263, 264). REDD1 is upregulated in mouse and human skin and in skeletal muscles following topical or oral GC administration. In mice, the expression of REDD1 is increased between 4 to 24 hours after treatment with topical GC (263) or after oral dexamethasone at 1 mg/kg (264). High levels of REDD1 correlate with reduced phosphorylation of downstream mammalian target of rapamycin complex-1 targets (Akt signaling pathway proteins S6 Ser^240/244^; 4E-BP1 Thr^37/46^, and ULK1 Ser^757^) resulting in reduction of protein synthesis (263, 264). In contrast, *REDD1* knockout mice did not demonstrate such downstream changes, and gene activation by GCs are altered in such a way that the therapeutic anti-inflammatory effects of GC were preserved. Thus, *REDD1* expression could represent a surrogate biomarker for GC signaling in skin or skeletal muscle; however, data is limited to only preclinical models receiving supraphysiological GC doses to date (240, 242).

### Bone Turnover Makers

The incidence of osteoporotic fractures may be as high as 20% in endogenous hypercortisolism and 30% to 50% in patients with long-term GC use (287). Prolonged exposure to GCs induces insulin resistance in bone cells resulting in increased bone marrow adiposity and bone fragility (288). Dose and duration of GC use influences fracture risk (289). Individuals receiving higher doses of oral prednisolone (≥7.5 mg per day) had a 2- to 2.5-fold increased fracture risk compared to those on lower doses (<2.5 mg per day) (289). Cumulative GC doses of >1 g over a year result in significant bone loss compared to those receiving lower cumulative GC doses (290). Interestingly, bone loss is more rapid in new GC users during the first year with bone density of up to 12% followed by a slower decline of 2% to 3% per year with persistent GC use (291).

GCs inhibit osteocalcin, which is a marker of osteoblast activity (266). In healthy subjects, 1 mg oral dexamethasone reduced osteocalcin levels from 28.3 ng/mL to 21.8 ng/mL at 8 hours (267). Likewise, intramuscular administration of 1 mg of ACTH at 2 Pm in healthy individuals (which induced a rise in cortisol to 58.3 mcg/dL, ie, 1608 nmol/L) reduced osteocalcin levels from 28.3 ng/mL to 12.5 ng/mL at 8 Am the following morning (267). In fact, GC administered in both oral (prednisolone 5 mg) or inhaled forms depresses osteocalcin levels acutely at 3 to 4 hours postdose with levels returning to pretreatment levels on tapering of GC doses (266, 268). A randomized cross-over study showed that healthy subjects (n = 37) receiving 7 days of treatment with prednisone at doses of 2.5 to 60 mg had a dose-dependent decrease in plasma osteocalcin levels as early as 4 hours postdose, which remained suppressed throughout the week (292).

The utility of serum osteocalcin as a biomarker has been explored in patients with CS. Morning serum osteocalcin levels were 21.5 ng/mL in healthy individuals and 8.35 ng/mL in overt CS, which corresponds to an AuROC of 0.923 in identifying individuals with CS (269). There was a negative correlation between osteocalcin levels and serum cortisol levels in patients with active CS in the morning (*r* = −0.39), at midnight (*r* = −0.54), and after low-dose dexamethasone suppression test (LDDST) (*r* = −0.53) (269). An increase in osteocalcin levels was also observed as early as 1 month after transsphenoidal surgery or adrenalectomy in patients with CS (270). While osteocalcin is helpful in validating the diagnosis of overt CS, its utility in identifying individuals with milder degrees of cortisol excess is yet to be evaluated. A recent retrospective study suggests osteocalcin could be a promising tool for identifying CS in postmenopausal women (age > 45 years) with osteoporosis (from those without CS) [AuROC: 0.960 (95% CI 0.892-1.000)] (293).

Other markers of bone formation, such as total and bone-specific alkaline phosphatase and procollagen type I carboxy-propeptide, are less sensitive to GC effects and display a milder and delayed decrease under GC therapy (268).

Bone resorption increases during GC treatment (294); however, data are inconsistent. C-terminal telopeptide of type 1 collagen (CTX) is a marker of osteoclastic activity and changes with cortisol levels but less sensitively than osteocalcin. Serum CTX levels correlate with morning (*r* +0.76) and postdexamethasone cortisol levels (*r* +0.43). CTX did not change in patients with CS; however, it was elevated in patients with ectopic ACTH (1.0 vs 0.4 ng/mL) who have higher cortisol levels (50 mcg/dL in ectopic ACTH CS vs 21 mcg/dL in other CS) (270). Overall, osteocalcin could present a relevant marker of GC status pending further study.

## Conclusion

GCs are essential hormones for the normal functioning of most organ systems in the body with both GC excess and GC deficiency leading to adverse health consequences. However, symptoms can be nonspecific, and reliance on cortisol levels alone as a readout of GC status can be unreliable. Dynamic tests used to make diagnoses are based on historical cut-off values and aim to detect more profound abnormalities in GC status. Moreover, cortisol values can be challenged due to a number of factors, including the pulsatile nature of cortisol secretion, interindividual variation in cortisol binding proteins, local tissue availability dictated by 11 β-HSD activity, and GC receptor sensitivity.

Downstream markers of GC action may provide an objective method to assess GC status. Such markers include those reflecting metabolomic and epigenetic changes. A comprehensive multiomic approach, applied in concert with serum GC levels, assessment of clinical symptoms and signs such as weight change and metabolic parameters, along with other markers of cortisol action, could enable a comprehensive and objective assessment of GC status in the future.

## References

[1] McMaster A, Jangani M, Sommer P, et al Ultradian cortisol pulsatility encodes a distinct, biologically important signal. PLoS One. 2011;6(1):e15766. 21267416 10.1371/journal.pone.0015766 PMC3022879

[2] Lightman SL, Birnie MT, Conway-Campbell BL. Dynamics of ACTH and cortisol secretion and implications for disease. Endocr Rev. 2020;41(3):bnaa002. 32060528 10.1210/endrev/bnaa002 PMC7240781

[3] Pivonello R, Isidori AM, De Martino MC, Newell-Price J, Biller BMK, Colao A. Complications of Cushing's syndrome: state of the art. Lancet Diabetes Endocrinol. 2016;4(7):611‐629. 27177728 10.1016/S2213-8587(16)00086-3

[4] Hahner S, Ross RJ, Arlt W, et al Adrenal insufficiency. Nat Rev Dis Primers. 2021;7(1):19. 33707469 10.1038/s41572-021-00252-7

[5] Faghih RT, Dahleh MA, Adler GK, Klerman EB, Brown EN. Deconvolution of serum cortisol levels by using compressed sensing. PLoS One. 2014;9(1):e85204. 24489656 10.1371/journal.pone.0085204 PMC3904842

[6] Lengton R, Iyer AM, Valk ES, et al Variation in glucocorticoid sensitivity and the relation with obesity. Obes Rev. 2022;23(3):e13401. 34837448 10.1111/obr.13401 PMC9285588

[7] Liyanarachchi K, Ross R, Debono M. Human studies on hypothalamo-pituitary-adrenal (HPA) axis. Best Pract Res Clin Endocrinol Metab. 2017;31(5):459‐473. 29223281 10.1016/j.beem.2017.10.011

[8] Mavroudis PD, Scheff JD, Calvano SE, Lowry SF, Androulakis IP. Entrainment of peripheral clock genes by cortisol. Physiol Genomics. 2012;44(11):607‐621. 22510707 10.1152/physiolgenomics.00001.2012 PMC3426436

[9] Charmandari E, Chrousos GP, Lambrou GI, et al Peripheral CLOCK regulates target-tissue glucocorticoid receptor transcriptional activity in a circadian fashion in man. PLoS One. 2011;6(9):e25612. 21980503 10.1371/journal.pone.0025612 PMC3182238

[10] Balbo M, Leproult R, Van Cauter E. Impact of sleep and its disturbances on hypothalamo-pituitary-adrenal axis activity. Int J Endocrinol. 2010;2010:759234. 20628523 10.1155/2010/759234 PMC2902103

[11] Weibel L, Spiegel K, Follenius M, Ehrhart J, Brandenberger G. Internal dissociation of the circadian markers of the cortisol rhythm in night workers. Am J Physiol. 1996;270(4 Pt 1):E608‐E613. 8928766 10.1152/ajpendo.1996.270.4.E608

[12] Roden M, Koller M, Pirich K, Vierhapper H, Waldhauser F. The circadian melatonin and cortisol secretion pattern in permanent night shift workers. Am J Physiol. 1993;265(1 Pt 2):R261‐R267. 8342696 10.1152/ajpregu.1993.265.1.R261

[13] Christ-Crain M, Kola B, Lolli F, et al AMP-activated protein kinase mediates glucocorticoid- induced metabolic changes: a novel mechanism in Cushing's syndrome. FASEB J. 2008;22(6):1672‐1683. 18198220 10.1096/fj.07-094144

[14] Bae YJ, Kratzsch J. Corticosteroid-binding globulin: modulating mechanisms of bioavailability of cortisol and its clinical implications. Best Pract Res Clin Endocrinol Metab. 2015;29(5):761‐772. 26522460 10.1016/j.beem.2015.09.001

[15] Meyer EJ, Nenke MA, Rankin W, Lewis JG, Torpy DJ. Corticosteroid-binding globulin: a review of basic and clinical advances. Horm Metab Res. 2016;48(6):359‐371. 27214312 10.1055/s-0042-108071

[16] Dichtel LE, Schorr M, Loures De Assis C, et al Plasma free cortisol in states of normal and altered binding globulins: implications for adrenal insufficiency diagnosis. J Clin Endocrinol Metab. 2019;104(10):4827‐4836. 31009049 10.1210/jc.2019-00022 PMC6735741

[17] Lewis J, Möpert B, Shand B, et al Plasma variation of corticosteroid-binding globulin and sex hormone-binding globulin. Horm Metab Res. 2006;38(4):241‐245. 16700005 10.1055/s-2006-925338

[18] Verbeeten KC, Ahmet AH. The role of corticosteroid-binding globulin in the evaluation of adrenal insufficiency. J Pediatr Endocrinol Metab. 2018;31(2):107‐115. 29194043 10.1515/jpem-2017-0270

[19] Hamrahian AH, Oseni TS, Arafah BM. Measurements of serum free cortisol in critically ill patients. N Engl J Med. 2004;350(16):1629‐1638. 15084695 10.1056/NEJMoa020266

[20] Wallace I, Cunningham S, Lindsay J. The diagnosis and investigation of adrenal insufficiency in adults. Ann Clin Biochem. 2009;46(Pt 5):351‐367. 19675057 10.1258/acb.2009.009101

[21] Klose MC, Lange M, Rasmussen AK, et al Factors influencing the adrenocorticotropin test: role of contemporary cortisol assays, body composition, and oral contraceptive agents. J Clin Endocrinol Metab. 2007;92(4):1326‐1333. 17244781 10.1210/jc.2006-1791

[22] Deuschle M, Gotthardt U, Schweiger U, et al With aging in humans the activity of the hypothalamus-pituitary-adrenal system increases and its diurnal amplitude flattens. Life Sci. 1997;61(22):2239‐2246. 9393943 10.1016/s0024-3205(97)00926-0

[23] Dmitrieva NO, Almeida DM, Dmitrieva J, Loken E, Pieper CF. A day-centered approach to modeling cortisol: diurnal cortisol profiles and their associations among U.S. adults. Psychoneuroendocrinology. 2013;38(10):2354‐2365. 23770247 10.1016/j.psyneuen.2013.05.003 PMC3776005

[24] Roelfsema F, van Heemst D, Iranmanesh A, Takahashi P, Yang R, Veldhuis JD. Impact of age, sex and body mass index on cortisol secretion in 143 healthy adults. Endocr Connect. 2017;6(7):500‐509. 28760748 10.1530/EC-17-0160 PMC5597974

[25] Van Cauter E, Leproult R, Kupfer DJ. Effects of gender and age on the levels and circadian rhythmicity of plasma cortisol. J Clin Endocrinol Metab. 1996;81(7):2468‐2473. 8675562 10.1210/jcem.81.7.8675562

[26] Almeida DM, Piazza JR, Stawski RS. Interindividual differences and intraindividual variability in the cortisol awakening response: an examination of age and gender. Psychol Aging. 2009;24(4):819‐827. 20025398 10.1037/a0017910 PMC2810505

[27] Karlamangla AS, Friedman EM, Seeman TE, Stawksi RS, Almeida DM. Daytime trajectories of cortisol: demographic and socioeconomic differences—findings from the National Study of Daily Experiences. Psychoneuroendocrinology. 2013;38(11):2585‐2597. 23831263 10.1016/j.psyneuen.2013.06.010 PMC3812359

[28] Walder DJ, Statucka M, Daly MP, Axen K, Haber M. Biological sex and menstrual cycle phase modulation of cortisol levels and psychiatric symptoms in a non-clinical sample of young adults. Psychiatry Res. 2012;197(3):314‐321. 22364929 10.1016/j.psychres.2011.09.009

[29] Montero-López E, Santos-Ruiz A, García-Ríos MC, Rodríguez-Blázquez M, Rogers HL, Peralta-Ramírez MI. The relationship between the menstrual cycle and cortisol secretion: daily and stress-invoked cortisol patterns. Int J Psychophysiol. 2018;131:67‐72. 29605399 10.1016/j.ijpsycho.2018.03.021

[30] Hamidovic A, Karapetyan K, Serdarevic F, Choi SH, Eisenlohr-Moul T, Pinna G. Higher circulating cortisol in the follicular vs. luteal phase of the menstrual cycle: a meta-analysis. Front Endocrinol (Lausanne). 2020;11:311. 32582024 10.3389/fendo.2020.00311 PMC7280552

[31] Kirschbaum C, Kudielka BM, Gaab J, Schommer NC, Hellhammer DH. Impact of gender, menstrual cycle phase, and oral contraceptives on the activity of the hypothalamus-pituitary-adrenal axis. Psychosom Med. 1999;61(2):154‐162. 10204967 10.1097/00006842-199903000-00006

[32] El-Farhan N, Pickett A, Ducroq D, et al Method-specific serum cortisol responses to the adrenocorticotrophin test: comparison of gas chromatography-mass spectrometry and five automated immunoassays. Clin Endocrinol (Oxf). 2013;78(5):673‐680. 22994849 10.1111/cen.12039

[33] El-Farhan N, Rees DA, Evans C. Measuring cortisol in serum, urine and saliva—are our assays good enough? Ann Clin Biochem. 2017;54(3):308‐322. 28068807 10.1177/0004563216687335

[34] Mattingly D . A simple fluorimetric method for the estimation of free 11-hydroxycorticoids in human plasma. J Clin Pathol. 1962;15(4):374‐379. 14471419 10.1136/jcp.15.4.374 PMC480416

[35] Campuzano HC, Wilkerson JE, Raven PB, Schabram T, Horvath SM. A radioimmunoassay for cortisol in human plasma. Biochem Med. 1973;7(3):350‐362. 4736696 10.1016/0006-2944(73)90056-2

[36] Bacarese-Hamilton T, Cattini R, Shandley C, Howard C, Palmer R, McFarthing K. A fully automated enzyme immunoassay for the measurement of cortisol in biological fluids. Eur J Clin Chem Clin Biochem. 1992;30(9):531‐535. 1457615 10.1515/cclm.1992.30.9.531

[37] Gatti R, Antonelli G, Prearo M, Spinella P, Cappellin E, De Palo EF. Cortisol assays and diagnostic laboratory procedures in human biological fluids. Clin Biochem. 2009;42(12):1205‐1217. 19414006 10.1016/j.clinbiochem.2009.04.011

[38] Aranda G, Careaga M, Hanzu FA, et al Accuracy of immunoassay and mass spectrometry urinary free cortisol in the diagnosis of Cushing's syndrome. Pituitary. 2016;19(5):496‐502. 27259502 10.1007/s11102-016-0730-5

[39] Casals G, Hanzu FA. Cortisol measurements in Cushing's syndrome: immunoassay or mass spectrometry? *Ann Lab Med*. 2020;40(4):285–296. 10.3343/alm.2020.40.4.285 PMC7054699 32067427

[40] Burt MG, Mangelsdorf BL, Rogers A, et al Free and total plasma cortisol measured by immunoassay and mass spectrometry following ACTH1–24 stimulation in the assessment of pituitary patients. J Clin Endocrinol Metab. 2013;98(5):1883‐1890. 23539724 10.1210/jc.2012-3576

[41] Tractenberg RE, Jonklaas J, Soldin SJ. Agreement of immunoassay and tandem mass spectrometry in the analysis of cortisol and free T4: interpretation and implications for clinicians. Int J Anal Chem. 2010;2010:234808. 20706537 10.1155/2010/234808 PMC2913524

[42] Choi MH . Clinical and technical aspects in free cortisol measurement. Endocrinol Metab. 2022;37(4):599‐607. 10.3803/EnM.2022.1549 PMC9449105 35982612

[43] Monaghan PJ, Owen LJ, Trainer PJ, Brabant G, Keevil BG, Darby D. Comparison of serum cortisol measurement by immunoassay and liquid chromatography-tandem mass spectrometry in patients receiving the 11 *β* -hydroxylase inhibitor metyrapone. Ann Clin Biochem. 2011;48(Pt 5):441‐446. 21813575 10.1258/acb.2011.011014

[44] Djedovic NK, Rainbow SJ. Detection of synthetic glucocorticoids by liquid chromatography-tandem mass spectrometry in patients being investigated for Cushing's syndrome. Ann Clin Biochem. 2011;48(Pt 6):542‐549. 21846739 10.1258/acb.2011.010250

[45] Marcos J, Renau N, Casals G, Segura J, Ventura R, Pozo OJ. Investigation of endogenous corticosteroids profiles in human urine based on liquid chromatography tandem mass spectrometry. Anal Chim Acta. 2014;812:92‐104. 24491769 10.1016/j.aca.2013.12.030

[46] Keevil BG . LC-MS/MS the first 20 years: a personal view. Ann Clin Biochem. 2022;59(1):3‐6. 34459220 10.1177/00045632211040059

[47] Hawley JM, Keevil BG. Endogenous glucocorticoid analysis by liquid chromatography–tandem mass spectrometry in routine clinical laboratories. J Steroid Biochem Mol Biol. 2016;162:27‐40. 27208627 10.1016/j.jsbmb.2016.05.014

[48] Perogamvros I, Ray DW, Trainer PJ. Regulation of cortisol bioavailability–effects on hormone measurement and action. Nat Rev Endocrinol. 2012;8(12):717‐727. 22890008 10.1038/nrendo.2012.134

[49] Pretorius CJ, Galligan JP, McWhinney BC, Briscoe SE, Ungerer JPJ. Free cortisol method comparison: ultrafiltation, equilibrium dialysis, tracer dilution, tandem mass spectrometry and calculated free cortisol. Clin Chim Acta. 2011;412(11-12):1043‐1047. 21334320 10.1016/j.cca.2011.02.019

[50] Ho JT, Al-Musalhi H, Chapman MJ, et al Septic shock and sepsis: a comparison of total and free plasma cortisol levels. J Clin Endocrinol Metab. 2006;91(1):105‐114. 16263835 10.1210/jc.2005-0265

[51] Fede G, Spadaro L, Tomaselli T, et al Comparison of total cortisol, free cortisol, and surrogate markers of free cortisol in diagnosis of adrenal insufficiency in patients with stable cirrhosis. Clin Gastroenterol Hepatol. 2014;12(3):504‐512.e8. 23978347 10.1016/j.cgh.2013.08.028

[52] Tan T, Chang L, Woodward A, et al Characterising adrenal function using directly measured plasma free cortisol in stable severe liver disease. J Hepatol. 2010;53(5):841‐848. 20739086 10.1016/j.jhep.2010.05.020

[53] Tordjman K, Jaffe A, Trostanetsky Y, Greenman Y, Limor R, Stern N. Low-dose (1 μg) adrenocorticotrophin (ACTH) stimulation as a screening test for impaired hypothalamo-pituitary-adrenal axis function: sensitivity, specificity and accuracy in comparison with the high-dose (250 μg) test: the low dose ACTH test for HPA assessment. Clin Endocrinol (Oxf). 2000;52(5):633‐640. 10792344 10.1046/j.1365-2265.2000.00984.x

[54] Reynolds RM, Stewart PM, Seckl JR, Padfield PL. Assessing the HPA axis in patients with pituitary disease: a UK survey. Clin Endocrinol (Oxf). 2006;64(1):82‐85. 16402933 10.1111/j.1365-2265.2005.02421.x

[55] Mackenzie SD, Gifford RM, Boyle LD, Crane MS, Strachan MWJ, Gibb FW. Validated criteria for the interpretation of a single measurement of serum cortisol in the investigation of suspected adrenal insufficiency. Clin Endocrinol (Oxf). 2019;91(5):608‐615. 31380575 10.1111/cen.14071

[56] Simpson H, Tomlinson J, Wass J, Dean J, Arlt W. Guidance for the prevention and emergency management of adult patients with adrenal insufficiency. Clin Med (Lond). 2020;20(4):371‐378. 32675141 10.7861/clinmed.2019-0324 PMC7385786

[57] Pearce SHS, Gan EH, Napier C. MANAGEMENT OF ENDOCRINE DISEASE: residual adrenal function in Addison's disease. Eur J Endocrinol. 2021;184(2):R61‐R67. 33306039 10.1530/EJE-20-0894 PMC7849375

[58] Debono M, Ross RJ. What is the best approach to tailoring hydrocortisone dose to meet patient needs in 2012? Clin Endocrinol (Oxf). 2013;78(5):659‐664. 23194144 10.1111/cen.12117

[59] Esposito D, Pasquali D, Johannsson G. Primary adrenal insufficiency: managing mineralocorticoid replacement therapy. J Clin Endocrinol Metab. 2018;103(2):376‐387. 29156052 10.1210/jc.2017-01928

[60] Bornstein SR, Allolio B, Arlt W, et al Diagnosis and treatment of primary adrenal insufficiency: an endocrine society clinical practice guideline. J Clin Endocrinol Metab. 2016;101(2):364‐389. 26760044 10.1210/jc.2015-1710 PMC4880116

[61] Pazderska A, Pearce SHs. Adrenal insufficiency—recognition and management. Clin Med. 2017;17(3):258‐262. 10.7861/clinmedicine.17-3-258 PMC6297573 28572228

[62] Martin-Grace J, Dineen R, Sherlock M, Thompson CJ. Adrenal insufficiency: physiology, clinical presentation and diagnostic challenges. Clin Chim Acta. 2020;505:78‐91. 32035851 10.1016/j.cca.2020.01.029

[63] Hahner S, Spinnler C, Fassnacht M, et al High incidence of adrenal crisis in educated patients with chronic adrenal insufficiency: a prospective study. J Clin Endocrinol Metab. 2015;100(2):407‐416. 25419882 10.1210/jc.2014-3191

[64] Dunlop D . Eighty-six cases of Addison's disease. Br Med J. 1963;2(5362):887‐891. 14067675 10.1136/bmj.2.5362.887 PMC1873052

[65] Erichsen MM, Løvås K, Skinningsrud B, et al Clinical, immunological, and genetic features of autoimmune primary adrenal insufficiency: observations from a Norwegian registry. J Clin Endocrinol Metab. 2009;94(12):4882‐4890. 19858318 10.1210/jc.2009-1368

[66] Ventura M, Serra-Caetano J, Cardoso R, et al The spectrum of pediatric adrenal insufficiency: insights from 34 years of experience. J Pediatr Endocrinol Metab. 2019;32(7):721‐726. 31194685 10.1515/jpem-2019-0030

[67] Husebye ES, Pearce SH, Krone NP, Kämpe O. Adrenal insufficiency. Lancet. 2021;397(10274):613‐629. 33484633 10.1016/S0140-6736(21)00136-7

[68] Braun LT, Riester A, Oßwald-Kopp A, et al Toward a diagnostic score in Cushing's syndrome. Front Endocrinol. 2019;10:766. 10.3389/fendo.2019.00766 PMC6856055 31787931

[69] Ilias I, Torpy DJ, Pacak K, Mullen N, Wesley RA, Nieman LK. Cushing's syndrome due to ectopic corticotropin secretion: twenty years’ experience at the National Institutes of Health. J Clin Endocrinol Metab. 2005;90(8):4955‐4962. 15914534 10.1210/jc.2004-2527

[70] Valassi E, Santos A, Yaneva M, et al The European registry on Cushing's syndrome: 2-year experience. Baseline demographic and clinical characteristics. Eur J Endocrinol. 2011;165(3):383‐392. 21715416 10.1530/EJE-11-0272

[71] van Aken MO, Pereira AM, Biermasz NR, et al Quality of life in patients after long-term biochemical cure of Cushing's disease. J Clin Endocrinol Metab. 2005;90(6):3279‐3286. 15741267 10.1210/jc.2004-1375

[72] Feelders R, Sharma S, Nieman L. Cushing's syndrome: epidemiology and developments in disease management. Clin Epidemiol. 2015;7:281‐293. 25945066 10.2147/CLEP.S44336 PMC4407747

[73] Lacroix A, Feelders RA, Stratakis CA, Nieman LK. Cushing's syndrome. Lancet. 2015;386(9996):913‐927. 26004339 10.1016/S0140-6736(14)61375-1

[74] Raff H, Carroll T. Cushing's syndrome: from physiological principles to diagnosis and clinical care. J Physiol. 2015;593(3):493‐506. 25480800 10.1113/jphysiol.2014.282871 PMC4324701

[75] Feelders RA, Pulgar SJ, Kempel A, Pereira AM. MANAGEMENT OF ENDOCRINE DISEASE: the burden of Cushing's disease: clinical and health-related quality of life aspects. Eur J Endocrinol. 2012;167(3):311‐326. 22728347 10.1530/EJE-11-1095

[76] Torpy DJ, Mullen N, Ilias I, Nieman LK. Association of hypertension and hypokalemia with Cushing's syndrome caused by ectopic ACTH secretion: a series of 58 cases. Ann N Y Acad Sci. 2002;970(1):134‐144. 12381548 10.1111/j.1749-6632.2002.tb04419.x

[77] Kazlauskaite R, Evans AT, Villabona CV, et al Corticotropin tests for hypothalamic-pituitary- adrenal insufficiency: a metaanalysis. J Clin Endocrinol Metab. 2008;93(11):4245‐4253. 18697868 10.1210/jc.2008-0710

[78] Endert E, Ouwehand A, Fliers E, Prummel MF, Wiersinga WM. Establishment of reference values for endocrine tests. Part IV: adrenal insufficiency. Neth J Med. 2005;63(11):435‐443. 16397312

[79] Fleseriu M, Hashim IA, Karavitaki N, et al Hormonal replacement in hypopituitarism in adults: an Endocrine Society Clinical Practice Guideline. J Clin Endocrinol Metab. 2016;101(11):3888‐3921. 27736313 10.1210/jc.2016-2118

[80] Javorsky BR, Raff H, Carroll TB, et al New cutoffs for the biochemical diagnosis of adrenal insufficiency after ACTH stimulation using specific cortisol assays. J Endocr Soc. 2021;5(4):bvab022. 33768189 10.1210/jendso/bvab022 PMC7975762

[81] Chitale A, Musonda P, McGregor AM, Dhatariya KK. Determining the utility of the 60 minutes cortisol measurement in the short synacthen test. Clin Endocrinol (Oxf). 2013;79(1):14‐19. 22747889 10.1111/j.1365-2265.2012.04478.x

[82] Cho HY, Kim JH, Kim SW, et al Different cut-off values of the insulin tolerance test, the high-dose short synacthen test (250 μg) and the low-dose short synacthen test (1 μg) in assessing central adrenal insufficiency. Clin Endocrinol (Oxf). 2014;81(1):77‐84. 24382108 10.1111/cen.12397

[83] Cegla J, Jones B, Seyani L, et al Comparison of the overnight metyrapone and glucagon stimulation tests in the assessment of secondary hypoadrenalism. Clin Endocrinol (Oxf). 2013;78(5):738‐742. 22998100 10.1111/cen.12043

[84] Berneis K, Staub JJ, Gessler A, Meier C, Girard J, Müller B. Combined stimulation of adrenocorticotropin and compound-S by single dose metyrapone test as an outpatient procedure to assess hypothalamic-pituitary-adrenal function. J Clin Endocrinol Metab. 2002;87(12):5470‐5475. 12466339 10.1210/jc.2001-011959

[85] Isidori AM, Kaltsas GA, Mohammed S, et al Discriminatory value of the low-dose dexamethasone suppression test in establishing the diagnosis and differential diagnosis of Cushing's syndrome. J Clin Endocrinol Metab. 2003;88(11):5299‐5306. 14602765 10.1210/jc.2003-030510

[86] Findling JW, Raff H, Aron DC. The low-dose dexamethasone suppression test: a reevaluation in patients with Cushing's syndrome. J Clin Endocrinol Metab. 2004;89(3):1222‐1226. 15001614 10.1210/jc.2003-030207

[87] Kyriazopoulou V, Vagenakis AG. Abnormal overnight dexamethasone suppression test in subjects receiving rifampicin therapy. J Clin Endocrinol Metab. 1992;75(1):315‐317. 1619024 10.1210/jcem.75.1.1619024

[88] Ceccato F, Barbot M, Zilio M, et al Screening tests for Cushing's syndrome: urinary free cortisol role measured by LC-MS/MS. J Clin Endocrinol Metab. 2015;100(10):3856‐3861. 26274344 10.1210/jc.2015-2507

[89] Nieman LK, Biller BMK, Findling JW, et al The diagnosis of Cushing's syndrome: an Endocrine Society Clinical Practice Guideline. J Clin Endocrinol Metab. 2008;93(5):1526‐1540. 18334580 10.1210/jc.2008-0125 PMC2386281

[90] Petersenn S, Newell-Price J, Findling JW, et al High variability in baseline urinary free cortisol values in patients with Cushing's disease. Clin Endocrinol (Oxf). 2014;80(2):261‐269. 23746264 10.1111/cen.12259 PMC4231220

[91] Raff H, Homar PJ, Burns EA. Comparison of two methods for measuring salivary cortisol. Clin Chem. 2002;48(1):207‐208. 11751565

[92] Mericq MV, Cutler GB Jr. High fluid intake increases urine free cortisol excretion in normal subjects. J Clin Endocrinol Metab. 1998;83(2):682‐684. 9467592 10.1210/jcem.83.2.4555

[93] Chan KCA, Lit LCW, Law ELK, et al Diminished urinary free cortisol excretion in patients with moderate and severe renal impairment. Clin Chem. 2004;50(4):757‐759. 15044334 10.1373/clinchem.2003.029934

[94] Newell-Price J, Trainer P, Perry L, Wass J, Grossman A, Besser M. A single sleeping midnight cortisol has 100% sensitivity for the diagnosis of Cushing's syndrome. Clin Endocrinol (Oxf). 1995;43(5):545‐550. 8548938 10.1111/j.1365-2265.1995.tb02918.x

[95] Reimondo G, Allasino B, Bovio S, Paccotti P, Angeli A, Terzolo M. Evaluation of the effectiveness of midnight serum cortisol in the diagnostic procedures for Cushing's syndrome. Eur J Endocrinol. 2005;153(6):803‐809. 16322385 10.1530/eje.1.02042

[96] Bergthorsdottir R, Leonsson-Zachrisson M, Odén A, Johannsson G. Premature mortality in patients with Addison's disease: a population-based study. J Clin Endocrinol Metab. 2006;91(12):4849‐4853. 16968806 10.1210/jc.2006-0076

[97] Müller L, Quinkler M. Imitating the cortisol profile improves the immune system. Nat Rev Endocrinol. 2018;14(3):137‐139. 29348474 10.1038/nrendo.2018.5

[98] Cain DW, Cidlowski JA. Immune regulation by glucocorticoids. Nat Rev Immunol. 2017;17(4):233‐247. 28192415 10.1038/nri.2017.1 PMC9761406

[99] Choudhury S, Tan T, Lazarus K, Meeran K. The use of prednisolone versus dual-release hydrocortisone in the treatment of hypoadrenalism. Endocr Connect. 2021;10(2):R66‐R76. 33449916 10.1530/EC-20-0473 PMC7983484

[100] Williams EL, Choudhury S, Tan T, Meeran K. Prednisolone replacement therapy mimics the circadian rhythm more closely than other glucocorticoids. J Appl Lab Med. 2016;1(2):152‐161. 33626793 10.1373/jalm.2016.020206

[101] Isidori A, Venneri M, Graziadio C, et al Effect of once-daily, modified-release hydrocortisone versus standard glucocorticoid therapy on metabolism and innate immunity in patients with adrenal. Lancet Diabetes Endocrinol. 2018;6(3):173‐185. 29229498 10.1016/S2213-8587(17)30398-4

[102] Choudhury S, Lightman S, Meeran K. Improving glucocorticoid replacement profiles in adrenal insufficiency. Clin Endocrinol (Oxf). 2019;91(3):367‐371. 31017681 10.1111/cen.13999

[103] Isidori AM, Pofi R, Hasenmajer V, Lenzi A, Pivonello R. Use of glucocorticoids in patients with adrenal insufficiency and COVID-19 infection. Lancet Diabetes Endocrinol. 2020;8(6):472‐473. 32334645 10.1016/S2213-8587(20)30149-2 PMC7180011

[104] Gagliardi L, Nenke MA, Thynne TRJ, et al Continuous subcutaneous hydrocortisone infusion therapy in Addison's disease: a randomized, placebo-controlled clinical trial. J Clin Endocrinol Metab. 2014;99(11):4149‐4157. 25127090 10.1210/jc.2014-2433

[105] Giraldi FP, Moro M, Cavagnini F. Gender-related differences in the presentation and course of Cushing's disease. J Clin Endocrinol Metab. 2003;88(4):1554‐1558. 12679438 10.1210/jc.2002-021518

[106] Newell-Price J, Bertagna X, Grossman AB, Nieman LK. Cushing's syndrome. Lancet. 2006;367(9522):1605‐1617. 16698415 10.1016/S0140-6736(06)68699-6

[107] Katz FH, Shannon IL. Parotid fluid cortisol and cortisone. J Clin Invest. 1969;48(5):848‐855. 4305375 10.1172/JCI106042 PMC322292

[108] Perogamvros I, Owen LJ, Newell-Price J, Ray DW, Trainer PJ, Keevil BG. Simultaneous measurement of cortisol and cortisone in human saliva using liquid chromatography–tandem mass spectrometry: application in basal and stimulated conditions. J Chromatogr B Analyt Technol Biomed Life Sci. 2009;877(29):3771‐3775. 10.1016/j.jchromb.2009.09.014 19783236

[109] Perogamvros I, Keevil BG, Ray DW, Trainer PJ. Salivary cortisone is a potential biomarker for serum free cortisol. J Clin Endocrinol Metab. 2010;95(11):4951‐4958. 20685855 10.1210/jc.2010-1215

[110] Debono M, Harrison RF, Whitaker MJ, et al Salivary cortisone reflects cortisol exposure under physiological conditions and after hydrocortisone. J Clin Endocrinol Metab. 2016;101(4):1469‐1477. 26812690 10.1210/jc.2015-3694

[111] Debono M, Elder CJ, Lewis J, et al Home waking salivary cortisone to screen for adrenal insufficiency. NEJM Evid. 2023;2(2):EVIDoa2200182. 10.1056/EVIDoa2200182 38320034

[112] Mohamed RS, Abuelgasim B, Barker S, et al Late-night salivary cortisol and cortisone should be the initial screening test for Cushing's syndrome. Endocr Connect. 2022;11(7):e220050. 35671282 10.1530/EC-22-0050 PMC9254321

[113] Blair J, Adaway J, Keevil B, Ross R. Salivary cortisol and cortisone in the clinical setting. Curr Opin Endocrinol Diabetes Obes. 2017;24(3):161‐168. 28375882 10.1097/MED.0000000000000328

[114] Raff H . Measurement of salivary cortisone to assess the adequacy of hydrocortisone replacement. J Clin Endocrinol Metab. 2016;101(4):1350‐1352. 26990943 10.1210/jc.2016-1228

[115] Harrison RF, Debono M, Whitaker MJ, Keevil BG, Newell-Price J, Ross RJ. Salivary cortisone to estimate cortisol exposure and sampling frequency required based on Serum cortisol measurements. J Clin Endocrinol Metab. 2019;104(3):765‐772. 30285244 10.1210/jc.2018-01172 PMC6349003

[116] Inder WJ, Dimeski G, Russell A. Measurement of salivary cortisol in 2012—laboratory techniques and clinical indications. Clin Endocrinol (Oxf). 2012;77(5):645‐651. 22812714 10.1111/j.1365-2265.2012.04508.x

[117] Miller R, Plessow F, Rauh M, Gröschl M, Kirschbaum C. Comparison of salivary cortisol as measured by different immunoassays and tandem mass spectrometry. Psychoneuroendocrinology. 2013;38(1):50‐57. 22641005 10.1016/j.psyneuen.2012.04.019

[118] Frugé AD, Cases MG, Howell CR, et al Fingernail and toenail clippings as a non-invasive measure of chronic cortisol levels in adult cancer survivors. Cancer Causes Control. 2018;29(1):185‐191. 29170880 10.1007/s10552-017-0989-5 PMC5764195

[119] van der Valk ES, Abawi O, Mohseni M, et al The relation between cortisol and anthropometric measurements throughout lifespan: a systematic review and meta-analysis. J Endocr Soc. 2021;5(Suppl 1):A30.

[120] Thomson S, Koren G, Fraser L-A, Rieder M, Friedman TC, Van Uum SHM. Hair analysis provides a historical record of cortisol levels in Cushing's syndrome. Exp Clin Endocrinol Diabetes. 2009;118(2):133‐138. 19609841 10.1055/s-0029-1220771 PMC2945912

[121] Noppe G, Van Rossum EFC, Vliegenthart J, Koper JW, Van Den Akker ELT. Elevated hair cortisol concentrations in children with adrenal insufficiency on hydrocortisone replacement therapy. Clin Endocrinol (Oxf). 2014;81(6):820‐825. 25039686 10.1111/cen.12551

[122] Staufenbiel SM, Andela CD, Manenschijn L, Pereira AM, Van Rossum EFC, Biermasz NR. Increased hair cortisol concentrations and BMI in patients with pituitary-adrenal disease on hydrocortisone replacement. J Clin Endocrinol Metab. 2015;100(6):2456‐2462. 25816049 10.1210/jc.2014-4328

[123] Manenschijn L, Koper JW, Van Den Akker ELT, et al A novel tool in the diagnosis and follow-up of (cyclic) Cushing's syndrome: measurement of long-term cortisol in scalp hair. J Clin Endocrinol Metab. 2012;97(10):E1836‐E1843. 22844063 10.1210/jc.2012-1852

[124] Gow R, Koren G, Rieder M, Van Uum S. Hair cortisol content in patients with adrenal insufficiency on hydrocortisone replacement therapy. Clin Endocrinol (Oxf). 2011;74(6):687‐693. 21521272 10.1111/j.1365-2265.2011.04001.x

[125] Liu CH, Doan SN. Innovations in biological assessments of chronic stress through hair and nail cortisol: conceptual, developmental, and methodological issues. Dev Psychobiol. 2019;61(3):465‐476. 30740655 10.1002/dev.21830 PMC6628692

[126] Chapman K, Holmes M, Seckl J. 11β-Hydroxysteroid dehydrogenases: intracellular gate-keepers of tissue glucocorticoid action. Physiol Rev. 2013;93(3):1139‐1206. 23899562 10.1152/physrev.00020.2012 PMC3962546

[127] Stimson RH, Andersson J, Andrew R, et al Cortisol release from adipose tissue by 11β-hydroxysteroid dehydrogenase type 1 in humans. Diabetes. 2009;58(1):46‐53. 18852329 10.2337/db08-0969 PMC2606892

[128] Basu R, Singh RJ, Basu A, et al Splanchnic cortisol production occurs in HumansEvidence for conversion of cortisone to cortisol via the 11-β hydroxysteroid dehydrogenase (11β-HSD) type 1 pathway. Diabetes. 2004;53(8):2051‐2059. 15277385 10.2337/diabetes.53.8.2051

[129] Harris HJ, Kotelevtsev Y, Mullins JJ, Seckl JR, Holmes MC. Intracellular regeneration of glucocorticoids by 11β-hydroxysteroid dehydrogenase (11β-HSD)-1 plays a key role in regulation of the hypothalamic-pituitary-adrenal axis: analysis of 11β-HSD-1-deficient mice*the wellcome trust supported this work through a program grant (to J.J.M. and J.R.S.) and a career development fellowship (to M.C.H.). Endocrinology. 2001;142(1):114‐120. 11145573 10.1210/endo.142.1.7887

[130] Masuzaki H, Paterson J, Shinyama H, et al A transgenic model of visceral obesity and the metabolic syndrome. Science. 2001;294(5549):2166‐2170. 11739957 10.1126/science.1066285

[131] Kotelevtsev Y, Holmes MC, Burchell A, et al 11β-Hydroxysteroid dehydrogenase type 1 knockout mice show attenuated glucocorticoid-inducible responses and resist hyperglycemia on obesity or stress. Proc Natl Acad Sci U S A. 1997;94(26):14924‐14929. 9405715 10.1073/pnas.94.26.14924 PMC25139

[132] Lindsay RS, Wake DJ, Nair S, et al Subcutaneous adipose 11β-hydroxysteroid dehydrogenase type 1 activity and messenger ribonucleic acid levels are associated with adiposity and insulinemia in Pima Indians and Caucasians. J Clin Endocrinol Metab. 2003;88(6):2738‐2744. 12788882 10.1210/jc.2002-030017

[133] Wake DJ, Rask E, Livingstone DEW, Söderberg S, Olsson T, Walker BR. Local and systemic impact of transcriptional up-regulation of 11β-hydroxysteroid dehydrogenase type 1 in adipose tissue in human obesity. J Clin Endocrinol Metab. 2003;88(8):3983‐3988. 12915696 10.1210/jc.2003-030286

[134] Tomlinson JW, Sinha B, Bujalska I, Hewison M, Stewart PM. Expression of 11beta-hydroxysteroid dehydrogenase type 1 in adipose tissue is not increased in human obesity. J Clin Endocrinol Metab. 2002;87(12):5630‐5635. 12466364 10.1210/jc.2002-020687

[135] Nair S, Lee YH, Lindsay RS, et al 11β-Hydroxysteroid dehydrogenase type 1: genetic polymorphisms are associated with type 2 diabetes in Pima Indians independently of obesity and expression in adipocyte and muscle. Diabetologia. 2004;47(6):1088‐1095. 15156315 10.1007/s00125-004-1407-6

[136] Draper N, Echwald SM, Lavery GG, et al Association studies between microsatellite markers within the gene encoding human 11beta-hydroxysteroid dehydrogenase type 1 and body mass index, waist to hip ratio, and glucocorticoid metabolism. J Clin Endocrinol Metab. 2002;87(11):4984‐4990. 12414862 10.1210/jc.2001-011375

[137] Tomlinson JW, Draper N, Mackie J, et al Absence of cushingoid phenotype in a patient with Cushing's disease due to defective cortisone to cortisol conversion. J Clin Endocrinol Metab. 2002;87(1):57‐62. 11788623 10.1210/jcem.87.1.8189

[138] Andrews RC, Rooyackers O, Walker BR. Effects of the 11 beta-hydroxysteroid dehydrogenase inhibitor carbenoxolone on insulin sensitivity in men with type 2 diabetes. J Clin Endocrinol Metab. 2003;88(1):285‐291. 12519867 10.1210/jc.2002-021194

[139] Rosenstock J, Banarer S, Fonseca VA, et al The 11-beta-hydroxysteroid dehydrogenase type 1 inhibitor INCB13739 improves hyperglycemia in patients with type 2 diabetes inadequately controlled by metformin monotherapy. Diabetes Care. 2010;33(7):1516‐1522. 20413513 10.2337/dc09-2315 PMC2890352

[140] Othonos N, Pofi R, Arvaniti A, et al 11β-HSD1 inhibition in men mitigates prednisolone-induced adverse effects in a proof-of-concept randomised double-blind placebo-controlled trial. Nat Commun. 2023;14(1):1025. 36823106 10.1038/s41467-023-36541-w PMC9950480

[141] Hughes KA, Webster SP, Walker BR. 11-Beta-hydroxysteroid dehydrogenase type 1 (11β-HSD1) inhibitors in type 2 diabetes mellitus and obesity. Expert Opin Investig Drugs. 2008;17(4):481‐496. 10.1517/13543784.17.4.481 18363514

[142] DeRijk RH, Schaaf M, De Kloet ER. Glucocorticoid receptor variants: clinical implications. J Steroid Biochem Mol Biol. 2002;81(2):103‐122. 12137800 10.1016/s0960-0760(02)00062-6

[143] Nicolaides NC, Chrousos G, Kino T. Glucocorticoid Receptor. Endotext 2000. Accessed April 17, 2022. https://www.ncbi.nlm.nih.gov/books/NBK279171/#!po=0.657895

[144] Ramamoorthy S, Cidlowski JA. Corticosteroids-mechanisms of action in health and disease. Rheum Dis Clin North Am. 2016;42(1):15‐31. 26611548 10.1016/j.rdc.2015.08.002 PMC4662771

[145] Sacta MA, Chinenov Y, Rogatsky I. Glucocorticoid signaling: an update from a genomic perspective. Annu Rev Physiol. 2016;78:155‐180. 26667074 10.1146/annurev-physiol-021115-105323

[146] Oakley RH, Cidlowski JA. The biology of the glucocorticoid receptor: new signaling mechanisms in health and disease. J Allergy Clin Immunol. 2013;132(5):1033‐1044. 24084075 10.1016/j.jaci.2013.09.007 PMC4084612

[147] Baker J, Ozsan I, Rodriguez Ospina S, Gulick D, Blair L. Hsp90 heterocomplexes regulate steroid hormone receptors: from stress response to psychiatric disease. Int J Mol Sci. 2018;20(1):79. 30585227 10.3390/ijms20010079 PMC6337637

[148] Vitellius G, Trabado S, Bouligand J, Delemer B, Lombès M. Pathophysiology of glucocorticoid signaling. Ann Endocrinol (Paris). 2018;79(3):98‐106. 29685454 10.1016/j.ando.2018.03.001

[149] van der Valk ES, Savas M, van Rossum EFC. Stress and obesity: are there more susceptible individuals? Curr Obes Rep. 2018;7(2):193‐203. 29663153 10.1007/s13679-018-0306-y PMC5958156

[150] Hawcutt DB, Francis B, Carr DF, et al Susceptibility to corticosteroid-induced adrenal suppression: a genome-wide association study. Lancet Respir Med. 2018;6(6):442‐450. 29551627 10.1016/S2213-2600(18)30058-4 PMC5971210

[151] Chruvattil R, Banerjee S, Nath S, et al Dexamethasone alters the appetite regulation via induction of hypothalamic insulin resistance in rat brain. Mol Neurobiol. 2017;54(9):7483‐7496. 27822713 10.1007/s12035-016-0251-2

[152] Uchoa ET, Aguilera G, Herman JP, Fiedler JL, Deak T, de Sousa MBC. Novel aspects of glucocorticoid actions. J Neuroendocrinol. 2014;26(9):557‐572. 24724595 10.1111/jne.12157 PMC4161987

[153] Valenzuela GA, Smalley WE, Schain DC, Vance ML, McCallum RW. Reversibility of gastric dysmotility in cortisol deficiency. Am J Gastroenterol. 1987;82(10):1066‐1068. 2821798

[154] Berthon BS, MacDonald-Wicks LK, Wood LG. A systematic review of the effect of oral glucocorticoids on energy intake, appetite, and body weight in humans. Nutr Res. 2014;34(3):179‐190. 24655484 10.1016/j.nutres.2013.12.006

[155] Di S, Malcher-Lopes R, Halmos K, Tasker JG. Nongenomic glucocorticoid inhibition via endocannabinoid release in the hypothalamus: a fast feedback mechanism. J Neurosci. 2003;23(12):4850‐4857. 12832507 10.1523/JNEUROSCI.23-12-04850.2003 PMC6741208

[156] van Schaik IN, Eftimov F, van Doorn PA, et al Pulsed high-dose dexamethasone versus standard prednisolone treatment for chronic inflammatory demyelinating polyradiculoneuropathy (PREDICT study): a double-blind, randomised, controlled trial. Lancet Neurol. 2010;9(3):245‐253. 20133204 10.1016/S1474-4422(10)70021-1

[157] Tataranni PA, Larson DE, Snitker S, Young JB, Flatt JP, Ravussin E. Effects of glucocorticoids on energy metabolism and food intake in humans. Am J Physiol. 1996;271(2 Pt 1):E317‐E325. 8770026 10.1152/ajpendo.1996.271.2.E317

[158] Bolton JL, Hayward C, Direk N, et al Genome wide association identifies common variants at the SERPINA6/SERPINA1 locus influencing plasma cortisol and corticosteroid binding globulin. PLoS Genet. 2014;10(7):e1004474. 25010111 10.1371/journal.pgen.1004474 PMC4091794

[159] Qi X, Cui B, Cao M. The role of morning plasma cortisol in obesity: a bidirectional Mendelian randomization study. J Clin Endocrinol Metab. 2022;107(5):e1954‐e1960. 35018462 10.1210/clinem/dgac008

[160] Chao AM, Jastreboff AM, White MA, Grilo CM, Sinha R. Stress, cortisol, and other appetite-related hormones: prospective prediction of 6-month changes in food cravings and weight. Obesity. 2017;25(4):713‐720. 28349668 10.1002/oby.21790 PMC5373497

[161] Hahner S, Loeffler M, Bleicken B, et al Epidemiology of adrenal crisis in chronic adrenal insufficiency: the need for new prevention strategies. Eur J Endocrinol. 2010;162(3):597‐602. 19955259 10.1530/EJE-09-0884

[162] Nerup J . Addison's disease—serological studies. Acta Endocrinol (Copenh). 1974;76(1):142‐158. 4208440 10.1530/acta.0.0760142

[163] Wong D, Siddall B, Wei W. Hormonal control of rat adrenal phenylethanolamine N-methyltransferase enzyme activity, the final critical pathway. Neuropsychopharmacology. 1995;13(3):223‐234. 8602895 10.1016/0893-133X(95)00066-M

[164] Fritz I, Levine R. Action of adrenal cortical steroids and nor-epinephrine on vascular responses of stress in adrenalectomized rats. Am J Physiol. 1951;165(2):456‐465. 14838134 10.1152/ajplegacy.1951.165.2.456

[165] Zuckerman-Levin N, Tiosano D, Eisenhofer G, Bornstein S, Hochberg Z. The importance of adrenocortical glucocorticoids for adrenomedullary and physiological response to stress: a study in isolated glucocorticoid deficiency. J Clin Endocrinol Metab. 2001;86(12):5920‐5924. 11739465 10.1210/jcem.86.12.8106

[166] Barbot M, Ceccato F, Scaroni C. The pathophysiology and treatment of hypertension in patients with Cushing's syndrome. Front Endocrinol. 2019;10:321. 10.3389/fendo.2019.00321 PMC6536607 31164868

[167] van der Valk ES, Smans LCCJ, Hofstetter H, et al Decreased physical activity, reduced QoL and presence of debilitating fatigue in patients with Addison's disease. Clin Endocrinol (Oxf). 2016;85(3):354‐360. 26953557 10.1111/cen.13059

[168] Alshekhlee A, Kaminski HJ, Ruff RL. Neuromuscular manifestations of endocrine disorders. Neurol Clin. 2002;20(1):35‐58. 11754301 10.1016/s0733-8619(03)00053-7

[169] Pereira RMR, de Carvalho JF. Glucocorticoid-induced myopathy. Joint Bone Spine. 2011;78(1):41‐44. 20471889 10.1016/j.jbspin.2010.02.025

[170] Batchelor TT, Taylor LP, Thaler HT, Posner JB, DeAngelis LM. Steroid myopathy in cancer patients. Neurology. 1997;48(5):1234‐1238. 9153449 10.1212/wnl.48.5.1234

[171] Lee HJ, Oran B, Saliba RM, et al Steroid myopathy in patients with acute graft-versus-host disease treated with high-dose steroid therapy. Bone Marrow Transplant. 2006;38(4):299‐303. 16819437 10.1038/sj.bmt.1705435

[172] Schakman O, Kalista S, Barbé C, Loumaye A, Thissen JP. Glucocorticoid-induced skeletal muscle atrophy. Int J Biochem Cell Biol. 2013;45(10):2163‐2172. 23806868 10.1016/j.biocel.2013.05.036

[173] Minetto MA, D’Angelo V, Arvat E, Kesari S. Diagnostic work-up in steroid myopathy. Endocrine. 2018;60(2):219‐223. 29143179 10.1007/s12020-017-1472-5

[174] Gupta A, Gupta Y. Glucocorticoid-induced myopathy: pathophysiology, diagnosis, and treatment. Indian J Endocrinol Metab. 2013;17(5):913‐916. 24083177 10.4103/2230-8210.117215 PMC3784879

[175] Gueugneau M, d’Hose D, Barbé C, et al Increased Serpina3n release into circulation during glucocorticoid-mediated muscle atrophy. J Cachexia Sarcopenia Muscle. 2018;9(5):929‐946. 29989354 10.1002/jcsm.12315 PMC6204594

[176] McPherron AC, Lawler AM, Lee SJ. Regulation of skeletal muscle mass in mice by a new TGF-p superfamily member. Nature. 1997;387(6628):83‐90. 9139826 10.1038/387083a0

[177] Zhang L, Rajan V, Lin E, et al Pharmacological inhibition of myostatin suppresses systemic inflammation and muscle atrophy in mice with chronic kidney disease. FASEB J. 2011;25(5):1653‐1663. 21282204 10.1096/fj.10-176917 PMC3079306

[178] Wang R, Jiao H, Zhao J, Wang X, Lin H. Glucocorticoids enhance muscle proteolysis through a myostatin-dependent pathway at the early stage. PLoS One. 2016;11(5):e0156225. 27227776 10.1371/journal.pone.0156225 PMC4882021

[179] Ma K, Mallidis C, Bhasin S, et al Glucocorticoid-induced skeletal muscle atrophy is associated with upregulation of myostatin gene expression. Am J Physiol Endocrinol Metab. 2003;285(2):363‐371. 10.1152/ajpendo.00487.2002 12721153

[180] Macedo AG, Krug ALO, Souza LM, et al Time-course changes of catabolic proteins following muscle atrophy induced by dexamethasone. Steroids. 2016;107:30‐36. 26730720 10.1016/j.steroids.2015.12.016

[181] Tsoriev TT, Belaya ZE, Rozhinskaya LY, et al Serum myokines levels in patients with endogenous cushing syndrome and acromegaly: cross-sectional case−control study. Ann Russ Acad Med Sci. 2016;71(3):240‐247. 10.15690/vramn659 29297640

[182] Inder WJ, Jang C, Obeyesekere VR, Alford FP. Dexamethasone administration inhibits skeletal muscle expression of the androgen receptor and IGF-1—implications for steroid-induced myopathy. Clin Endocrinol (Oxf). 2010;73(1):126‐132. 19681914 10.1111/j.1365-2265.2009.03683.x

[183] Musarò A, Giacinti C, Borsellino G, et al Stem cell-mediated muscle regeneration is enhanced by local isoform of insulin-like growth factor 1. Proc Natl Acad Sci U S A. 2004;101(5):1206‐1210. 14745025 10.1073/pnas.0303792101 PMC337031

[184] Latres E, Amini AR, Amini AA, et al Insulin-like growth factor-1 (IGF-1) inversely regulates atrophy-induced genes via the phosphatidylinositol 3-kinase/Akt/mammalian target of rapamycin (PI3K/Akt/mTOR) pathway. J Biol Chem. 2005;280(4):2737‐2744. 15550386 10.1074/jbc.M407517200

[185] Li B-G, Hasselgren P-O, Fang C-H. Insulin-like growth factor-I inhibits dexamethasone-induced proteolysis in cultured L6 myotubes through PI3 K/Akt/GSK-3β and PI3K/Akt/mTOR-dependent mechanisms. Int J Biochem Cell Biol. 2005;37(10):2207‐2216. 15927518 10.1016/j.biocel.2005.04.008

[186] Zapf J, Hauri C, Waldvogel M, Froesch ER. Acute metabolic effects and half-lives of intravenously administered insulinlike growth factors I and II in normal and hypophysectomized rats. J Clin Invest. 1986;77(6):1768‐1775. 3711334 10.1172/JCI112500 PMC370532

[187] Tanaka N, Ryoke T, Hongo M, et al Effects of growth hormone and IGF-I on cardiac hypertrophy and gene expression in mice. Am J Physiol. 1998;275(2):H393‐H399. 9683425 10.1152/ajpheart.1998.275.2.H393

[188] Miell JP, Taylor AM, Jones J, et al The effects of dexamethasone treatment on immunoreactive and bioactive insulin-like growth factors (IGFs) and IGF-binding proteins in normal male volunteers. J Endocrinol. 1993;136(3):525‐533. 7682595 10.1677/joe.0.1360525

[189] Ramshanker N, Aagaard M, Hjortebjerg R, et al Effects of prednisolone on serum and tissue fluid IGF-I receptor activation and post-receptor signaling in humans. J Clin Endocrinol Metab. 2017;102(11):4031‐4040. 28945869 10.1210/jc.2017-00696

[190] Tauber RN, Camic CL, Zhang S, Chomentowski PJ 3rd. Comparison of multi-frequency bioelectrical impedance and dual-energy X-ray absorptiometry to assess body composition in college-aged adults. Int J Exerc Sci. 2020;13(4):1595‐1604. 33414874 10.70252/LMVI3697 PMC7745892

[191] Moon JR, Stout JR, Smith-Ryan AE, et al Tracking fat-free mass changes in elderly men and women using single-frequency bioimpedance and dual-energy X-ray absorptiometry: a four-compartment model comparison. Eur J Clin Nutr. 2013;67(Suppl 1):S40‐S46. 23299870 10.1038/ejcn.2012.163

[192] Fragala MS, Kenny AM, Kuchel GA. Muscle quality in aging: a multi-dimensional approach to muscle functioning with applications for treatment. Sports Med. 2015;45(5):641‐658. 25655372 10.1007/s40279-015-0305-z

[193] Wang L, Yin L, Zhao Y, et al Muscle density, but not size, correlates well with muscle strength and physical performance. J Am Med Dir Assoc. 2021;22(4):751‐759.e2. 32768372 10.1016/j.jamda.2020.06.052

[194] Miller BS, Ignatoski KM, Daignault S, et al A quantitative tool to assess degree of sarcopenia objectively in patients with hypercortisolism. Surgery. 2011;150(6):1178‐1185. 22136838 10.1016/j.surg.2011.09.020

[195] Khaleeli AA, Betteridge DJ, Edwards RHT, Round JM, Ross EJ. Effect of treatment of cushing's syndrome on skeletal muscle structure and function. Clin Endocrinol (Oxf). 1983;19(4):547‐556. 6627703 10.1111/j.1365-2265.1983.tb00030.x

[196] Minetto MA, Caresio C, Salvi M, et al Ultrasound-based detection of glucocorticoid-induced impairments of muscle mass and structure in Cushing's disease. J Endocrinol Invest. 2019;42(7):757‐768. 30443856 10.1007/s40618-018-0979-9

[197] Minetto MA, Lanfranco F, Botter A, et al Do muscle fiber conduction slowing and decreased levels of circulating muscle proteins represent sensitive markers of steroid myopathy? A pilot study in Cushing's disease. Eur J Endocrinol. 2011;164(6):985‐993. 21402749 10.1530/EJE-10-1169

[198] Blijham PJ, Hengstman GJD, Hama-Amin AD, van Engelen BGM, Zwarts MJ. Needle electromyographic findings in 98 patients with myositis. Eur Neurol. 2006;55(4):183‐188. 16772711 10.1159/000093866

[199] Palve SS, Palve SB. Impact of aging on nerve conduction velocities and late responses in healthy individuals. J Neurosci Rural Pract. 2018;9(1):112‐116. 29456354 10.4103/jnrp.jnrp_323_17 PMC5812134

[200] Olnes MJ, Kotliarov Y, Biancotto A, et al Effects of systemically administered hydrocortisone on the human immunome. Sci Rep. 2016;6:23002. 26972611 10.1038/srep23002 PMC4789739

[201] Tresoldi AS, Sumilo D, Perrins M, et al Increased infection risk in Addison's disease and congenital adrenal hyperplasia. J Clin Endocrinol Metab. 2020;105(2):418‐429. 31532828 10.1210/clinem/dgz006 PMC7046014

[202] Bensing S, Brandt L, Tabaroj F, et al Increased death risk and altered cancer incidence pattern in patients with isolated or combined autoimmune primary adrenocortical insufficiency. Clin Endocrinol (Oxf). 2008;69(5):697‐704. 18727712 10.1111/j.1365-2265.2008.03340.x

[203] Edvardsen K, Bjånesøy T, Hellesen A, et al Peripheral blood cells from patients with autoimmune Addison's disease poorly respond to interferons in vitro, despite elevated serum levels of interferon-inducible chemokines. J Interferon Cytokine Res. 2015;35(10):759‐770. 25978633 10.1089/jir.2014.0171 PMC4589105

[204] Kovacs WJ . To B or not to B? Glucocorticoid impact on B lymphocyte fate and function. Endocrinology. 2014;155(2):339‐342. 24446731 10.1210/en.2013-2085

[205] Hasenmajer V, Sbardella E, Sciarra F, Minnetti M, Isidori AM, Venneri MA. The immune system in Cushing's syndrome. Trends Endocrinol Metab. 2020;31(9):655‐669. 32387195 10.1016/j.tem.2020.04.004

[206] Barahona M-J, Sucunza N, Resmini E, et al Persistent body fat mass and inflammatory marker increases after long-term cure of Cushing's syndrome. J Clin Endocrinol Metab. 2009;94(9):3365‐3371. 19509101 10.1210/jc.2009-0766

[207] Li J-X, Cummins CL. Fresh insights into glucocorticoid-induced diabetes mellitus and new therapeutic directions. Nat Rev Endocrinol. 2022;18(9):540‐557. 35585199 10.1038/s41574-022-00683-6 PMC9116713

[208] Giorgino F, Almahfouz A, Goodyear LJ, Smith RJ. Glucocorticoid regulation of insulin receptor and substrate IRS-1 tyrosine phosphorylation in rat skeletal muscle in vivo. J Clin Invest. 1993;91(5):2020‐2030. 7683695 10.1172/JCI116424 PMC288200

[209] Saad MJ, Folli F, Kahn JA, Kahn CR. Modulation of insulin receptor, insulin receptor substrate-1, and phosphatidylinositol 3-kinase in liver and muscle of dexamethasone-treated rats. J Clin Invest. 1993;92(4):2065‐2072. 7691892 10.1172/JCI116803 PMC288376

[210] Ruzzin J, Wagman AS, Jensen J. Glucocorticoid-induced insulin resistance in skeletal muscles: defects in insulin signalling and the effects of a selective glycogen synthase kinase-3 inhibitor. Diabetologia. 2005;48(10):2119‐2130. 16078016 10.1007/s00125-005-1886-0

[211] Weinstein SP, Wilson CM, Pritsker A, Cushman SW. Dexamethasone inhibits insulin-stimulated recruitment of GLUt4 to the cell surface in rat skeletal muscle. Metab Clin Exp. 1998;47(1):3‐6. 10.1016/s0026-0495(98)90184-6 9440469

[212] Löfberg E, Gutierrez A, Wernerman J, et al Effects of high doses of glucocorticoids on free amino acids, ribosomes and protein turnover in human muscle. Eur J Clin Invest. 2002;32(5):345‐353. 12027875 10.1046/j.1365-2362.2002.00993.x

[213] Short KR, Bigelow ML, Nair KS. Short-term prednisone use antagonizes insulin's anabolic effect on muscle protein and glucose metabolism in young healthy people. Am J Physiol Endocrinol Metab. 2009;297(6):E1260‐E1268. 19738036 10.1152/ajpendo.00345.2009 PMC2793048

[214] Xu C, He J, Jiang H, et al Direct effect of glucocorticoids on lipolysis in adipocytes. Mol Endocrinol. 2009;23(8):1161‐1170. 19443609 10.1210/me.2008-0464 PMC5419195

[215] Rafacho A, Cestari TM, Taboga SR, Boschero AC, Bosqueiro JR. High doses of dexamethasone induce increased β-cell proliferation in pancreatic rat islets. Am J Physiol Endocrinol Metab. 2009;296(4):E681‐E689. 19158320 10.1152/ajpendo.90931.2008

[216] Sato S, Saisho Y, Inaishi J, et al Effects of glucocorticoid treatment on β- and α-cell mass in Japanese adults with and without diabetes. Diabetes. 2015;64(8):2915‐2927. 25883114 10.2337/db15-0151

[217] van Raalte DH, Nofrate V, Bunck MC, et al Acute and 2-week exposure to prednisolone impair different aspects of β-cell function in healthy men. Eur J Endocrinol. 2010;162(4):729‐735. 20124412 10.1530/EJE-09-1034

[218] Wise JK, Hendler R, Felig P. Influence of glucocorticoids on glucagon secretion and plasma amino acid concentrations in man. J Clin Invest. 1973;52(11):2774‐2782. 4748510 10.1172/JCI107473 PMC302545

[219] Larsson H, Ahrén B. Insulin resistant subjects lack islet adaptation to short-term dexamethasone-induced reduction in insulin sensitivity. Diabetologia. 1999;42(8):936‐943. 10491753 10.1007/s001250051251

[220] Kola B, Christ-Crain M, Lolli F, et al Changes in adenosine 5′-monophosphate-activated protein kinase as a mechanism of visceral obesity in Cushing's syndrome. J Clin Endocrinol Metab. 2008;93(12):4969‐4973. 18782871 10.1210/jc.2008-1297 PMC7611639

[221] Carling D . AMPK signalling in health and disease. Curr Opin Cell Biol. 2017;45:31‐37. 28232179 10.1016/j.ceb.2017.01.005

[222] Nader N, Ng SSM, Lambrou GI, et al AMPK regulates metabolic actions of glucocorticoids by phosphorylating the glucocorticoid receptor through p38 MAPK. Mol Endocrinol. 2010;24(9):1748‐1764. 20660302 10.1210/me.2010-0192 PMC2940476

[223] Pernicova I, Kelly S, Ajodha S, et al Metformin to reduce metabolic complications and inflammation in patients on systemic glucocorticoid therapy: a randomised, double-blind, placebo-controlled, proof-of-concept, phase 2 trial. Lancet Diabetes Endocrinol. 2020;8(4):278‐291. 32109422 10.1016/S2213-8587(20)30021-8

[224] Campbell JE, Peckett AJ, D'souza AM, Hawke TJ, Riddell MC. Adipogenic and lipolytic effects of chronic glucocorticoid exposure. Am J Physiol Cell Physiol. 2011;300(1):C198‐C209. 20943959 10.1152/ajpcell.00045.2010

[225] Koliwad SK, Kuo T, Shipp LE, et al Angiopoietin-like 4 (ANGPTL4, fasting-induced adipose factor) is a direct glucocorticoid receptor target and participates in glucocorticoid-regulated triglyceride metabolism. J Biol Chem. 2009;284(38):25593‐25601. 19628874 10.1074/jbc.M109.025452 PMC2757961

[226] Djurhuus CB, Gravholt CH, Nielsen S, et al Effects of cortisol on lipolysis and regional interstitial glycerol levels in humans. Am J Physiol Endocrinol Metab. 2002;283(1):E172‐E177. 12067858 10.1152/ajpendo.00544.2001

[227] Hazlehurst JM, Gathercole LL, Nasiri M, et al Glucocorticoids fail to cause insulin resistance in human subcutaneous adipose tissue in vivo. J Clin Endocrinol Metab. 2013;98(4):1631‐1640. 23426618 10.1210/jc.2012-3523

[228] Pilkis SJ, Granner DK. Molecular physiology of the regulation of hepatic gluconeogenesis and glycolysis. Annu Rev Physiol. 1992;54:885‐909. 1562196 10.1146/annurev.ph.54.030192.004321

[229] Geer EB, Islam J, Buettner C. Mechanisms of glucocorticoid-induced insulin resistance. Endocrinol Metab Clin North Am. 2014;43(1):75‐102. 24582093 10.1016/j.ecl.2013.10.005 PMC3942672

[230] Ibrahim MM . Subcutaneous and visceral adipose tissue: structural and functional differences. Obes Rev. 2010;11(1):11‐18. 19656312 10.1111/j.1467-789X.2009.00623.x

[231] Rockall A, Sohaib S, Evans D, et al Computed tomography assessment of fat distribution in male and female patients with Cushing's syndrome. Eur J Endocrinol. 2003;149(6):561‐567. 14640998 10.1530/eje.0.1490561

[232] Stimson RH, Anderson AJ, Ramage LE, et al Acute physiological effects of glucocorticoids on fuel metabolism in humans are permissive but not direct. Diabetes Obes Metab. 2017;19(6):883‐891. 28177189 10.1111/dom.12899 PMC5484992

[233] Faggiano A, Pivonello R, Spiezia S, et al Cardiovascular risk factors and common carotid artery caliber and stiffness in patients with Cushing's disease during active disease and 1 year after disease remission. J Clin Endocrinol Metab. 2003;88(6):2527‐2533. 12788849 10.1210/jc.2002-021558

[234] Hayashi R, Tamada D, Murata M, et al Glucocorticoid replacement affects serum adiponectin levels and HDL-C in patients with secondary adrenal insufficiency. J Clin Endocrinol Metab. 2019;104(12):5814‐5822. 31290981 10.1210/jc.2019-00420

[235] Werumeus Buning J, Dimova LG, Perton FG, Tietge UJF, van Beek AP, Dullaart RPF. Downregulation of cholesteryl ester transfer protein by glucocorticoids: a randomised study on HDL. Eur J Clin Invest. 2017;47(7):494‐503. 28542805 10.1111/eci.12770

[236] Wang X, Magkos F, Patterson BW, Reeds DN, Kampelman J, Mittendorfer B. Low-dose dexamethasone administration for 3 weeks favorably affects plasma HDL concentration and composition but does not affect very low-density lipoprotein kinetics. Eur J Endocrinol. 2012;167(2):217‐223. 22619349 10.1530/EJE-12-0180 PMC3638974

[237] Filipsson H, Monson JP, Koltowska-Häggström M, Mattsson A, Johannsson G. The impact of glucocorticoid replacement regimens on metabolic outcome and comorbidity in hypopituitary patients. J Clin Endocrinol Metab. 2006;91(10):3954‐3961. 16895963 10.1210/jc.2006-0524

[238] Lewandowski KC, Szosland K, Lewinski A. Short-term dexamethasone administration does not alter serum adiponectin or resistin concentrations in overweight and obese subjects despite an increase in insulin resistance. Clin Endocrinol (Oxf). 2006;65(4):551‐552. 16984253 10.1111/j.1365-2265.2006.02638.x

[239] Fallo F, Scarda A, Sonino N, et al Effect of glucocorticoids on adiponectin: a study in healthy subjects and in Cushing's syndrome. Eur J Endocrinol. 2004;150(3):339‐344. 15012619 10.1530/eje.0.1500339

[240] Newcomer JW, Selke G, Melson AK, Gross J, Vogler GP, Dagogo-Jack S. Dose-dependent cortisol-induced increases in plasma leptin concentration in healthy humans. Arch Gen Psychiatry. 1998;55(11):995. 9819068 10.1001/archpsyc.55.11.995

[241] Weise M, Abad V, Considine RV, Nieman L, Rother KI. Leptin secretion in Cushing's syndrome: preservation of diurnal rhythm and absent response to corticotropin-releasing hormone. J Clin Endocrinol Metab. 1999;84(6):2075‐2079. 10372713 10.1210/jcem.84.6.5773

[242] Cizza G, Lotsikas AJ, Licinio J, Gold PW, Chrousos GP. Plasma leptin levels do not change in patients with Cushing's disease shortly after correction of hypercortisolism. J Clin Endocrinol Metab. 1997;82(8):2747‐2750. 9253364 10.1210/jcem.82.8.4139

[243] Widjaja A, Schürmeyer TH, Von Zur Mühlen A, Brabant G. Determinants of serum leptin levels in Cushing's syndrome. J Clin Endocrinol Metab. 1998;83(2):600‐603. 9467580 10.1210/jcem.83.2.4566

[244] Ebrecht M, Buske-Kirschbaum A, Hellhammer D, et al Tissue specificity of glucocorticoid sensitivity in healthy adults. J Clin Endocrinol Metab. 2000;85(10):3733‐3739. 11061532 10.1210/jcem.85.10.6891

[245] Kachroo P, Stewart ID, Kelly RS, et al Metabolomic profiling reveals extensive adrenal suppression due to inhaled corticosteroid therapy in asthma. Nat Med. 2022;28(4):814‐822. 35314841 10.1038/s41591-022-01714-5 PMC9350737

[246] Prete A, Subramanian A, Bancos I, et al Cardiometabolic disease burden and steroid excretion in benign adrenal tumors: a cross-sectional multicenter study. Ann Intern Med. 2022;175(3):325‐334. 34978855 10.7326/M21-1737

[247] Espiard S, McQueen J, Sherlock M, et al Improved urinary cortisol metabolome in addison disease: a prospective trial of dual-release hydrocortisone. J Clin Endocrinol Metab. 2021;106(3):814‐825. 33236103 10.1210/clinem/dgaa862 PMC7947853

[248] Chantzichristos D, Svensson P-A, Garner T, et al Identification of human glucocorticoid response markers using integrated multi-omic analysis from a randomized crossover trial. eLife. 2021;10:e62236. 33821793 10.7554/eLife.62236 PMC8024021

[249] Vetrivel S, Zhang R, Engel M, et al Circulating microRNA expression in Cushing's syndrome. Front Endocrinol. 2021;12:620012. 10.3389/fendo.2021.620012 PMC7937959 33692756

[250] Lockhart SM, Saudek V, O’Rahilly S. GDF15: a hormone conveying somatic distress to the brain. Endocr Rev. 2020;41(4):bnaa007. 32310257 10.1210/endrev/bnaa007 PMC7299427

[251] Melvin A, Chantzichristos D, Kyle CJ, et al GDF15 is elevated in conditions of glucocorticoid deficiency and is modulated by glucocorticoid replacement. J Clin Endocrinol Metab. 2020;105(5):1427‐1434. 31853550 10.1210/clinem/dgz277 PMC7105349

[252] Gutierrez LS, Lopez-Dee Z, Pidcock K. Thrombospondin-1: multiple paths to inflammation. Mediators Inflamm. 2011;2011: 296069. 21765615 10.1155/2011/296069 PMC3134184

[253] Lawler JW, Slayter HS, Coligan JE. Isolation and characterization of a high molecular weight glycoprotein from human blood platelets. J Biol Chem. 1978;253(23):8609‐8616. 101549

[254] Barclay JL, Petersons CJ, Keshvari S, et al Thrombospondin-1 is a glucocorticoid responsive protein in humans. Eur J Endocrinol. 2016;174(2):193‐201. 26578641 10.1530/EJE-15-0964

[255] Inder WJ, Mohamed A, Keshvari S, et al Ex vivo glucocorticoid-induced secreted proteome approach for discovery of glucocorticoid-responsive proteins in human serum. Proteomics Clin Appl. 2021;15(2-3):e2000078. 33641263 10.1002/prca.202000078

[256] Cesana-Nigro N, Keshvari S, Barclay JL, et al The effect of glucocorticoids on thrombospondin-1, osteocalcin and the thrombospondin-1:osteocalcin ratio in humans. Clin Endocrinol (Oxf). 2019;91(6):728‐736. 31612515 10.1111/cen.14108

[257] Bancos I, Hatipoglu BA, Yuen KCJ, Chandramohan L, Chaudhari S, Moraitis AG. Evaluation of FKBP5 as a cortisol activity biomarker in patients with ACTH-dependent Cushing syndrome. J Clin Transl Endocrinol. 2021;24:100256. 34258233 10.1016/j.jcte.2021.100256 PMC8260880

[258] Zannas AS, Wiechmann T, Gassen NC, Binder EB. Gene–stress–epigenetic regulation of FKBP5: clinical and translational implications. Neuropsychopharmacology. 2016;41(1):261‐274. 26250598 10.1038/npp.2015.235 PMC4677131

[259] Gallo LI, Lagadari M, Piwien-Pilipuk G, Galigniana MD. The 90-kDa heat-shock protein (Hsp90)-binding immunophilin FKBP51 is a mitochondrial protein that translocates to the nucleus to protect cells against oxidative stress. J Biol Chem. 2011;286(34):30152‐30160. 21730050 10.1074/jbc.M111.256610 PMC3191054

[260] Sævik ÅB, Wolff AB, Björnsdottir S, et al Potential transcriptional biomarkers to guide glucocorticoid replacement in autoimmune Addison's disease. J Endocr Soc. 2021;5(3):bvaa202. 33553982 10.1210/jendso/bvaa202 PMC7853175

[261] Bali U, Phillips T, Hunt H, Unitt J. FKBP5 mRNA expression is a biomarker for GR antagonism. J Clin Endocrinol Metab. 2016;101(11):4305‐4312. 27459525 10.1210/jc.2016-1624

[262] Winkler BK, Lehnert H, Oster H, Kirchner H, Harbeck B. FKBP5 methylation as a possible marker for cortisol state and transient cortisol exposure in healthy human subjects. Epigenomics. 2017;9(10):1279‐1286. 28875708 10.2217/epi-2017-0057

[263] Baida G, Bhalla P, Kirsanov K, et al REDD1 functions at the crossroads between the therapeutic and adverse effects of topical glucocorticoids. EMBO Mol Med. 2015;7(1):42‐58. 25504525 10.15252/emmm.201404601 PMC4309667

[264] Britto FA, Begue G, Rossano B, et al REDD1 deletion prevents dexamethasone-induced skeletal muscle atrophy. Am J Physiol Endocrinol Metab. 2014;307(11):E983‐E993. 25315696 10.1152/ajpendo.00234.2014

[265] Kumari R, Willing LB, Jefferson LS, Simpson IA, Kimball SR. REDD1 (regulated in development and DNA damage response 1) expression in skeletal muscle as a surrogate biomarker of the efficiency of glucocorticoid receptor blockade. Biochem Biophys Res Commun. 2011;412(4):644‐647. 21856283 10.1016/j.bbrc.2011.08.017 PMC3175593

[266] Meeran K, Hattersley A, Burrin J, Shiner R, Ibbertson K. Oral and inhaled corticosteroids reduce bone formation as shown by plasma osteocalcin levels. Am J Respir Crit Care Med. 1995;151(2 Pt 1):333‐336. 7842187 10.1164/ajrccm.151.2.7842187

[267] Majnik J, Szücs N, Patócs A, et al Effect of single doses of dexamethasone and adrenocorticotrop hormone on serum bone markers in healthy subjects and in patients with adrenal incidentalomas and Cushing's syndrome. J Endocrinol Invest. 2004;27(8):747‐753. 15636428 10.1007/BF03347517

[268] Nielsen HK, Charles P, Mosekilde L. The effect of single oral doses of prednisone on the circadian rhythm of serum osteocalcin in normal subjects. J Clin Endocrinol Metab. 1988;67(5):1025‐1030. 3263379 10.1210/jcem-67-5-1025

[269] Sereg M, Tke J, Patócs A, et al Diagnostic performance of salivary cortisol and serum osteocalcin measurements in patients with overt and subclinical Cushing's syndrome. Steroids. 2011;76(1-2):38‐42. 20813120 10.1016/j.steroids.2010.08.007

[270] Szappanos Á, Toke J, Lippai D, et al Bone turnover in patients with endogenous Cushing's syndrome before and after successful treatment. Osteoporos Int. 2010;21(4):637‐645. 19513576 10.1007/s00198-009-0978-y

[271] Prete A, Subramanian A, Bancos I, et al Cardiometabolic disease burden and steroid excretion in benign adrenal tumors. Ann Intern Med. 2022;175(3):325‐334. 34978855 10.7326/M21-1737

[272] Varadarajan S, Breda C, Smalley JL, et al The transrepression arm of glucocorticoid receptor signaling is protective in mutant huntingtin-mediated neurodegeneration. Cell Death Differ. 2015;22(8):1388‐1396. 25656655 10.1038/cdd.2015.1 PMC4495362

[273] Cortez MA, Bueso-Ramos C, Ferdin J, Lopez-Berestein G, Sood AK, Calin GA. MicroRNAs in body fluids—the mix of hormones and biomarkers. Nat Rev Clin Oncol. 2011;8(8):467‐477. 21647195 10.1038/nrclinonc.2011.76 PMC3423224

[274] Dai C, Zhang Y, Xu Z, Jin M. MicroRNA-122-5p inhibits cell proliferation, migration and invasion by targeting CCNG1 in pancreatic ductal adenocarcinoma. Cancer Cell Int. 2020;20:98. 32256207 10.1186/s12935-020-01185-z PMC7106816

[275] Choi K-Y, Kim D-B, Kim M-J, et al Higher plasma thrombospondin-1 levels in patients with coronary artery disease and diabetes mellitus. Korean Circ J. 2012;42(2):100‐106. 22396697 10.4070/kcj.2012.42.2.100 PMC3291719

[276] Hayden K, Tetlow L, Byrne G, Bundred N. Radioimmunoassay for the measurement of thrombospondin in plasma and breast cyst fluid: validation and clinical application. Ann Clin Biochem. 2000;37(3):319‐325. 10817245 10.1258/0004563001899212

[277] Leclerc N, Luppen CA, Ho VV, et al Gene expression profiling of glucocorticoid-inhibited osteoblasts. J Mol Endocrinol. 2004;33(1):175‐193. 15291752 10.1677/jme.0.0330175

[278] Rae M, Mohamad A, Price D, et al Cortisol inactivation by 11β-hydroxysteroid dehydrogenase-2 may enhance endometrial angiogenesis via reduced thrombospondin-1 in heavy menstruation. J Clin Endocrinol Metab. 2009;94(4):1443‐1450. 19158196 10.1210/jc.2008-1879

[279] Wochnik GM, Rüegg J, Abel GA, Schmidt U, Holsboer F, Rein T. FK506-binding proteins 51 and 52 differentially regulate dynein interaction and nuclear translocation of the glucocorticoid receptor in mammalian cells. J Biol Chem. 2005;280(6):4609‐4616. 15591061 10.1074/jbc.M407498200

[280] Berger SL, Kouzarides T, Shiekhattar R, Shilatifard A. An operational definition of epigenetics. Genes Dev. 2009;23(7):781‐783. 19339683 10.1101/gad.1787609 PMC3959995

[281] Buitrago D, Labrador M, Arcon JP. et al. Impact of DNA methylation on 3D genome structure. *Nat Commun* 2021;12:3243 10.1038/s41467-021-23142-8 PMC8163762 34050148

[282] Klengel T, Mehta D, Anacker C, et al Allele-specific FKBP5 DNA demethylation mediates gene–childhood trauma interactions. Nat Neurosci. 2013;16(1):33‐41. 23201972 10.1038/nn.3275 PMC4136922

[283] Matsuda A, Asada Y, Takakuwa K, Sugita J, Murakami A, Ebihara N. DNA methylation analysis of human trabecular meshwork cells during dexamethasone stimulation. Invest Opthalmol Vis Sci. 2015;56(6):3801‐3809. 10.1167/iovs.14-16008 26066748

[284] Lee RS, Tamashiro KLK, Yang X, et al A measure of glucocorticoid load provided by DNA methylation of fkbp5 in mice. Psychopharmacology (Berl). 2011;218(1):303‐312. 21509501 10.1007/s00213-011-2307-3 PMC3918452

[285] Resmini E, Santos A, Aulinas A, et al Reduced DNA methylation of FKBP5 in Cushing's syndrome. Endocrine. 2016;54(3):768‐777. 27664120 10.1007/s12020-016-1083-6 PMC6391874

[286] Canal M, Romaní-Aumedes J, Martín-Flores N, Pérez-Fernández V, Malagelada C. RTP801/REDD1: a stress coping regulator that turns into a troublemaker in neurodegenerative disorders. Front Cell Neurosci. 2014;8:313. 25324725 10.3389/fncel.2014.00313 PMC4183088

[287] Amiche MA, Albaum JM, Tadrous M, et al Fracture risk in oral glucocorticoid users: a Bayesian meta-regression leveraging control arms of osteoporosis clinical trials. Osteoporos Int. 2016;27(5):1709‐1718. 26694595 10.1007/s00198-015-3455-9

[288] Sadie-Van Gijsen H, Crowther NJ, Hough FS, Ferris WF. The interrelationship between bone and fat: from cellular see-saw to endocrine reciprocity. Cell Mol Life Sci. 2013;70(13):2331‐2349. 23178849 10.1007/s00018-012-1211-2 PMC11113730

[289] Van Staa TP, Leufkens HGM, Abenhaim L, Zhang B, Cooper C. Use of oral corticosteroids and risk of fractures. J Bone Miner Res. 2000;15(6):993‐1000. 10841167 10.1359/jbmr.2000.15.6.993

[290] van Staa TP, Leufkens HGM, Cooper C. The epidemiology of corticosteroid-induced osteoporosis: a meta-analysis. Osteoporos Int. 2002;13(10):777‐787. 12378366 10.1007/s001980200108

[291] LoCascio V, Bonucci E, Imbimbo B, et al Bone loss in response to long-term glucocorticoid therapy. Bone Miner. 1990;8(1):39‐51. 2306553 10.1016/0169-6009(91)90139-q

[292] Fleishaker DL, Mukherjee A, Whaley FS, Daniel S, Zeiher BG. Safety and pharmacodynamic dose response of short-term prednisone in healthy adult subjects: a dose ranging, randomized, placebo-controlled, crossover study. BMC Musculoskelet Disord. 2016;17:293. 27424036 10.1186/s12891-016-1135-3 PMC4947329

[293] Belaya ZE, Iljin AV, Melnichenko GA, et al Diagnostic performance of osteocalcin measurements in patients with endogenous Cushing's syndrome. Bonekey Rep. 2016;5:815. 27347399 10.1038/bonekey.2016.42 PMC4909045

[294] Dempster DW . Perspectives bone histomorphometry in glucocorticoid-induced osteoporosis. J Bone Miner Res. 1989;4(2):137‐141. 2658477 10.1002/jbmr.5650040202

